# Phosphatidylcholine biosynthesis pathways in *Cryptococcus neoformans*: functional interplay and impact on virulence

**DOI:** 10.3389/fcimb.2025.1736171

**Published:** 2026-01-27

**Authors:** Filipe dos S. Timboni, Aisel Valle Garay, Raffael J. Araújo de Castro, Gabrielly Bindo Trindade, Namuhell Oliveira da Silva, Luísa Coutinho Coelho, Vitoria Merçon Dias, Ana Paula Campos Vieira de Sousa, Maurizio Del Poeta, Patrícia Albuquerque, Anamélia Lorenzetti Bocca, Larissa Fernandes

**Affiliations:** 1 Postgraduate Program of Microbial Biology, University of Brasília, Brasília, Federal District, Brazil; 2 Department of Cell Biology, Institute of Biological Sciences, University of Brasília, Brasília, Federal District, Brazil; 3 Laboratory of Molecular Biophysics, University of Brasília, Brasília, Federal District, Brazil; 4 Department of Genetics and Morphology, Institute of Biological Sciences, University of Brasília, Brasília, Federal District, Brazil; 5 Department of Biochemistry and Immunology, Ribeirão Preto Medical School, University from São Paulo, Ribeirão Preto, São Paulo, Brazil; 6 Bi−Institutional Translational Medicine Platform, Oswaldo Cruz Foundation (Fiocruz), Ribeirão Preto, São Paulo, Brazil; 7 Laboratory of Molecular Biology of Pathogenic Fungi, Institute of Biological Sciences, University of Brasília, Brasília, Federal District, Brazil; 8 Center of Molecular Biotechnology (C-BIOTECH), University of Brasília, Brasília, Federal District, Brazil; 9 Postgraduate Program of Molecular Biology, University of Brasília, Brasília, Federal District, Brazil; 10 Faculty of Health Sciences and Technologies, University of Brasilia, Brasília, Federal District, Brazil; 11 Department of Microbiology and Immunology, Stony Brook University, Stony Brook, NY, United States; 12 Division of Infectious Diseases, School of Medicine, Stony Brook University, Stony Brook, NY, United States; 13 Veterans Affairs Medical Center, Northport, NY, United States; 14 National Institute of Science and Technology in Human Pathogenic Fungi, Ribeirão Preto, São Paulo, Brazil

**Keywords:** *Cryptococcus neoformans*, *de novo* pathway, Kennedy pathway, phosphatidylcholine biosynthesis, virulence, α-glycerophosphorylcholine

## Abstract

As fungal diseases emerge, new studies aim to understand how different metabolic pathways, including the biosynthesis of phospholipids, influence the fungal pathogenicity. Therefore, to investigate the role of phosphatidylcholine (PC) in the biology of the human fungal pathogen *Cryptococcus neoformans*, a double mutant lacking *OPI3* (phosphatidylethanolamine methyltransferase) from the *de novo* pathway, and *PCT1* (choline phosphate cytidylyltransferase) from the salvage Kennedy pathway was generated using *double-joint* PCR coupled with biolistic technique for gene deletion. Phenotypic and virulence assays were performed, including growth viability in minimal nutrient, melanization, capsule expansion and titanization, lipid droplet analysis and *in vivo* infection in larval and murine models. The double mutant (*opi3Δpct1Δ*) exhibited normal growth in complex medium, but displayed severe growth defects and loss of viability under nutrient-limited conditions. Supplementation with L-α-glycerophosphorylcholine (GPC), PC or sorbitol fully restores growth, suggesting compensation of GPC-dependent reacylation pathway. Disruption of PC biosynthesis affected important virulence traits, including capsule formation, melanization, and titan cell development, and increased susceptibility to membrane stresses. In vivo, in both the *Galleria mellonella* and murine models, *opi3Δpct1Δ* was hypovirulent with reduced brain colonization. Other studies with *C. neoformans* and *Candida albicans*, another pathogenic yeast, showed no impact in deletion of either *OPI3* or *PCT1* alone for virulence and pathogenicity. Therefore, these findings highlight the critical role of PC biosynthesis for maintaining membrane integrity, morphological plasticity and host dissemination of *C. neoformans*.

## 1 Introduction

*Cryptococcus neoformans* is a basidiomycete fungus, ubiquitously distributed in the environment, commonly associated with trees, decaying wood, and bird guano. Its spores spread through the air and infect the lungs of mammals, including humans. After the initial pulmonary infection, the disease can spread to the central nervous system (CNS) (Nielsen et al., 2005; Kwon-Chung et al., 2014). In 2020 alone, cryptococcal meningitis (CM) caused over 110,000 deaths globally, with most cases occurring in individuals with compromised immune systems, especially those living with acquired immunodeficiency syndrome (AIDS) (Zhao et al., 2023). Moreover, the rise of antifungal drug-resistant strains and the growing population of immunosuppressed individuals, such as organ transplant recipients and patients undergoing chemotherapy, have contributed to making cryptococcosis a significant public health concern (Pyrgos et al., 2013; Zhao et al., 2023). Consequently, *C. neoformans* has been classified as a critical priority pathogen by the World Health Organization (WHO, 2022).

Fungal diseases have emerged as significant global health threats, and the limited availability of effective treatment options has intensified the need for new therapeutic strategies. Advances in molecular tools have enabled the study of the biosynthesis and organization of key cellular components such as the plasma membrane by elucidating the roles of various proteins and lipids in its structure and function. Among these molecules, phospholipids, the primary constituents of plasma and organelle membranes, have attracted considerable attention in fungal pathogens (Lösel, 1990; Kodedová et al., 2019; Li et al., 2025). Phospholipids modulate membrane properties (Wang and Tontonoz, 2019), while also serving as critical mediators of intracellular signaling in various cellular processes (Zhang and Majerus, 1998). These molecules are not only essential for membrane integrity and cellular function, but they also represent promising targets for antifungal therapy. Therefore, understanding the roles of phospholipids and their biosynthetic pathways is crucial for deciphering the mechanisms of fungal virulence and pathogenicity (van Meer et al., 2008; Sant et al., 2016; Pan et al., 2018).

**Figure 1:**
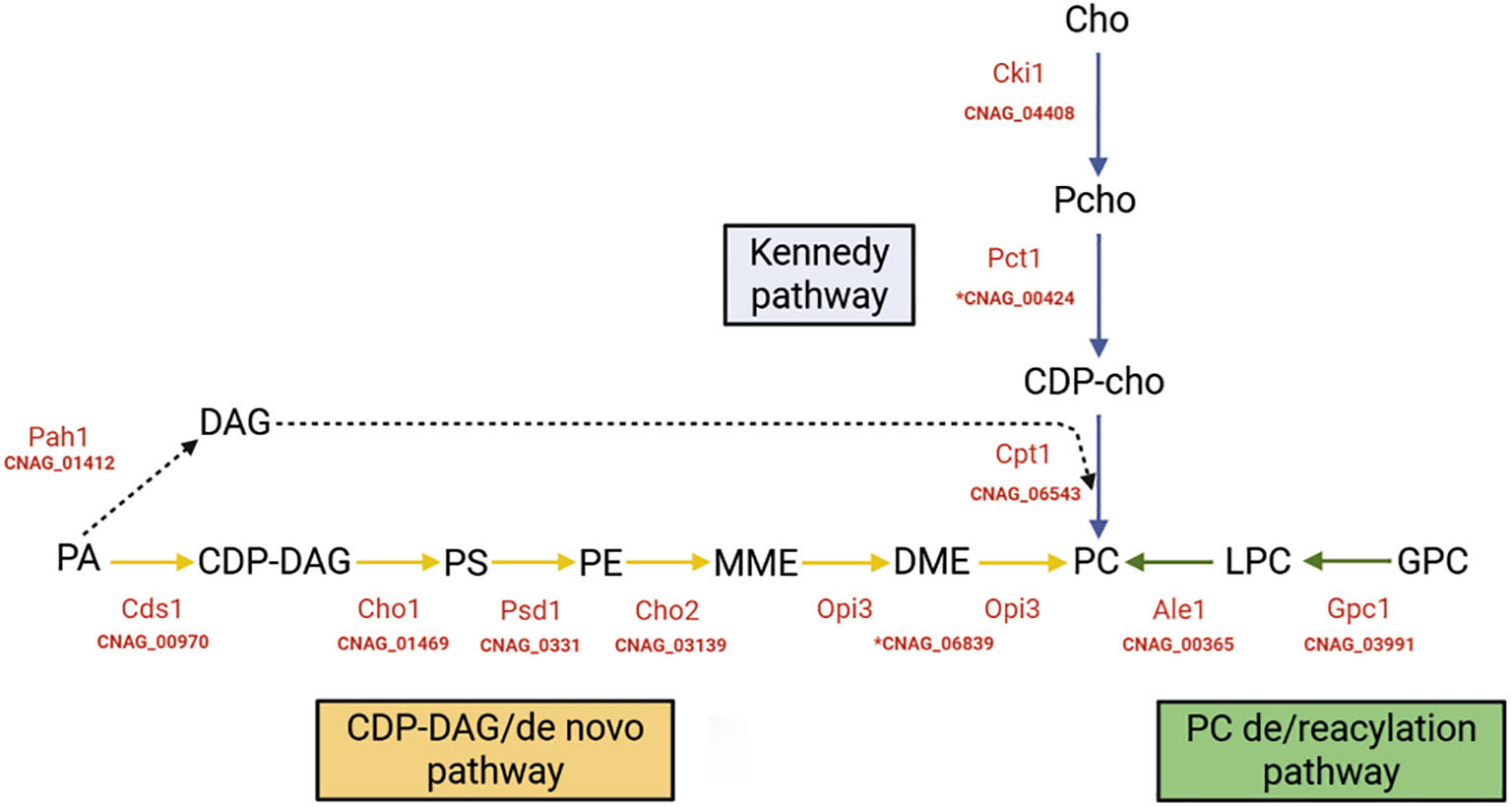
PC synthesis pathways and related genes in *C. neoformans.* PC biosynthesis known main pathways and enzymes associated in red. Phosphatidic acid (PA), synthesized from glycerol-3-phosphate is the precursor molecule for the synthesis of cytidine diphosphate diacylglycerol (CDP-DAG) and diacylglycerol (DAG). CDP-DAG is the main precursor of the *de novo* biosynthetic pathway of phosphatidylserine (PS), phosphatidylethanolamine (PE) and phosphatidylcholine (PC) (represented by yellow arrows). DAG is used in the final reactions of the salvage Kennedy pathway (dashed black arrow), which utilizes free choline for the biosynthesis of PC (blue arrows). The accessory deacylation/reacylation pathway (green arrows) synthesizes PC from the acylation of α-glycerophosphorylcholine (GPC) by the enzyme Gpc1. MME, monomethyl phosphatidylethanolamine. DME, dimethylphosphatidylethanolamine. Cho, choline. PCho, phosphocholine. CDP-Cho, cytidine diphosphocholine. LPC, lysophosphatidylcholine. In parentheses are the CNAG codes used in this study, which refer to the genomic sequences available in the H99 Genome Project database at the Broad Institute. CNAG codes preceded by * were used for mutant construction in this study. Image created by author.

Phosphatidylcholine (PC) is the most abundant phospholipid in eukaryotic cells, regulating membrane fluidity, permeability, and curvature (McMaster, 2018). It also participates in vesicular trafficking and organelle biogenesis (Howe and McMaster, 2001). In fungi, PC can be synthesized through three metabolic pathways, including (i) the *de novo* pathway mediated by the methyltransferases Cho2 and Opi3 (Carman and Han, 2009); (ii) the Kennedy pathway which incorporates exogenous choline through the cytidylyltransferase Pct1 (Kent, 2005; Gibellini and Smith, 2010) and (iii) the glycerophosphorylcholine reacylation pathway, which utilizes L-α-glycerophosphorylcholine (GPC) to generate PC through specific acyltransferases (Głąb et al., 2016; Anaokar et al., 2019) (**Figure 1**). The latter route, evolutionarily conserved in fungi and plants but absent in mammals, offers a unique opportunity for selective antifungal intervention (Głąb et al., 2016).

While the *de novo* and Kennedy pathways are well-characterized in fungi, their functional interplay and redundancy remain not fully understood. In *Saccharomyces cerevisiae*, deletion of *OPI3* disrupts lipid homeostasis and triggers an unfolded protein response, while the Kennedy pathway compensates by recycling exogenous choline (Carman and Henry, 1999; Boumann et al., 2006). In *Candida albicans*, perturbations in PE methyltransferases affect dimorphic transitions, and *PCT1* disruption attenuates virulence (Davis et al., 2018; Tams et al., 2019). In *Fusarium graminearum*, deletion of *CHO2* and *OPI3* impairs growth under nutrient-limited conditions, alters colony morphology, and reduces virulence in wheat infection (Wang et al., 2019). In *C. neoformans*, *OPI3* deletion modifies membrane composition, capsule formation, and cell wall architecture; however, virulence remains intact, likely due to metabolic compensation via the Kennedy pathway and the assimilation of host-derived choline (Lee et al., 2024). Dissecting the contribution of each PC synthesis pathway is therefore critical to understanding how this pathogen maintains membrane homeostasis and adapts to host environments.

We hypothesized that simultaneous disruption of the *de novo* (*OPI3*) and Kennedy (*PCT1*) pathways would severely compromise PC biosynthesis, leading to defects in membrane stability, morphological plasticity, and virulence. To test this, we generated a double mutant (*opi3Δpct1Δ*) and performed phenotypic and infection assays. Our findings reveal that *C. neoformans* relies on PC biosynthesis for survival under nutrient-limited conditions and for the expression of key virulence traits, including capsule formation, melanization, and titan cell development. Furthermore, disruption of both pathways markedly reduced fungal dissemination to the central nervous system, highlighting the central role of PC metabolism in pathogenesis. Together, these findings establish PC biosynthesis as a core physiological determinant governing fungal physiology and virulence, particularly in *C. neoformans*.

## 2 Materials and methods

### 2.1 Strains and growth conditions

The fungal strains used in this study (**Table 1**) were stored at -80°C in tubes containing a mixture of 75% YPD and 25% glycerol. Unless otherwise specified, strains were retrieved from the storage every seven days and cultured on solid YPD plates (1% yeast extract, 2% peptone, 2% dextrose, 1.5% agar, pH 5.6) at 30°C for 48 to 72 hours. Prior to each experiment, colonies from each strain were used to inoculate liquid YPD and incubate overnight at 30°C under constant agitation. For flow cytometry, lipid extraction and *in vivo* assays, the cultured strains were washed three times in sterile saline and suspensions of 10^7^ CFU/mL were inoculated into liquid minimal medium (MM) composed of Dextrose 15 mM, MgSO_4–_10 mM, Glycine 13 mM, KH_2_PO_4_ 29.4 mM, and Thiamine 3 µM (pH 5.5). Cultures were incubated at 30°C under agitation for 15 hours before use.

**Table 1:** *C. neoformans* strains used in this study.

Strain	Description and FungiDB code	Origin
KN99α	Wild-type (WT)	Madhani collection
*opi3Δ*	Mutant deleted for *OPI3* (CNAG_06839)	Madhani collection
*pct1Δ*	Mutant deleted for *PCT1* (CNAG_00424)	Constructed in this study
*opi3Δpct1Δ*	Mutant deleted for *OPI3* and *PCT1* (CNAG_00424 and CNAG_06839)	Constructed in this study
*pct1Δ*::NEO::*PCT1*	Reconstituted *pct1Δ* + *PCT1*	Constructed in this study
*opi3Δ*::NEO::*OPI3*	Reconstituted *opi3Δ* + *OPI3*	Constructed in this study
*opi3Δpct1Δ*::NEO::*PCT1*	Reconstituted *opi3Δpct1Δ* + *PCT1*	Constructed in this study
*opi3Δpct1Δ*::NEO::*OPI3*	Reconstituted *opi3Δpct1Δ* + *OPI3*	Constructed in this study

### 2.2 Deletion of *PCT1* gene and double mutant construction

The annotated sequence of the choline-phosphate cytidylyltransferase (*PCT1*) gene from the *C. neoformans* H99 genome was obtained from the FungiDB database (http://fungidb.org). Based on the nucleotide sequence CNAG_00424 (*Cn*PCT1), primers for polymerase chain reaction (PCR) were designed using the Primer3 tool (https://fungidb.org/fungidb/app) (**Supplementary Table 1**), for gene deletion in the parental strain and in *CnOPI3* deleted mutant (obtained from Madhani’s knock out collection). Gene deletion was carried out using double joint-PCR (DJ-PCR) method for selection cassette construction, as described by Kim et al (Kim et al., 2009). (**Supplementary Figure 1**), followed by biolistic transformation for genomic integration. The selection cassette was generated using plasmid pPZP-HYG2, which carries *HPH*, (conferring hygromycin resistance) under control of *C. neoformans* actin promoter and *TRPC* terminator (Walton et al., 2005).

Mutant strains reconstitutions were performed via co-transformation using PCR-amplified *PCT1* or *OPI3* gene fragments in combination with pJAF1 plasmid. This plasmid contains the neomycin resistance marker, *Neo^R^*, driven by *C. neoformans* actin promoter and terminator (Fraser et al., 2003). Confirmation of gene deletions or reconstitutions were performed by selection marker screening and diagnostic PCRs (**Supplementary Figures 1, 2**).

### 2.3 Phenotypic characterization assays

The growth rate of each mutant was assessed in liquid YPD medium (Yeast 1%, peptone 2%, dextrose 2%, pH 5.6) and in liquid MM, either supplemented or not with 10µM choline chloride (Sigma), 10µM L-α-glycerophosphorylcholine (Sigma), 500 µM phosphatidylcholine (Sigma) or 10% fetal bovine serum. Wild-type and mutant cells were inoculated at a final cell density of 5 × 10^4^ cells/mL into the respective media. Then, 200 μL of each inoculum was dispensed into wells of a 96-well microplate. Each experiment was realized in technical duplicates or triplicates and two biological replicates. The plates were incubated at 30°C or 37°C in a microplate spectrophotometer (Epoch 1, Gen5 software v.3.11, Biotek), under agitation at 190 rpm for 96 to 120 hours. Optical density at 600 nm (OD 600 nm) was recorded every 30 minutes.

Susceptibility to various stressors was assessed by preparing a starting inoculum of 2 × 10^8^ CFU/mL, which was then serially diluted to a final concentration of 2 × 10² CFU/mL. A volume of 5 μL from each dilution was spotted onto YPD or MM agar plates supplemented with different stress-inducing agents: Congo Red (0.5%), Calcofluor White (1.5 mg/mL), sodium chloride (1 M), sodium nitrite (4 mg/mL hydrogen peroxide (2 mM), sodium dodecyl sulfate (0.01%), and/or sorbitol (2 M). Plates were incubated at 30°C and photographed after colony formation.

The melanization phenotype was evaluated following a protocol adapted from Eisenman (Eisenman et al., 2011). A total of 1 × 10^7^ cells from each strain were inoculated onto MM agar plates supplemented with 1 mM L-DOPA (Sigma-Aldrich), with or without 1 mM choline chloride or 10 µM GPC. The plates were incubated at 30°C and 37°C for one week protected from light. Photographs were taken at defined time intervals: 24 h, 48 h, 72 h, and 96 h. To quantify melanization, mean gray values of the colonies were analyzed using ImageJ v.1.53r software (https://imagej.net/ij/). Statistical analysis was performed using two-way ANOVA with Tukey’s correction for multiple comparisons. Data represents the average of two biological replicates.

Polysaccharide capsule size and titan cell formation was evaluated as described by Hommel et al (Hommel et al., 2018). Briefly, 1 × 10^6^ cells were inoculated into liquid MM and incubated at 30°C for 20 hours under agitation. Then, 1 mL of the culture was centrifuged and resuspended in India ink for optical microscopy analysis. Slides were prepared with 5 μL of the cell suspension mixed with India ink and imaged using a Nikon ECLIPSE Si microscope with a RoHS, BC1200 camera under a 40 × objective. The capsular diameter of 100 cells per strain was measured by subtracting the cell body diameter from the total diameter. The diameter of 200 cells per strain was also measured and titan cells were defined as those with ≥10 μm. All measurements were conducted using the image analysis and processing software ImageJ v.1.53r. Statistical analysis was performed using Kruskal-Wallis test with Tukey’s corrections for multiple comparisons. Three biological replicates were performed.

Cell viability in nutrient-poor medium was evaluated by incubating previously grown strains in liquid MM at a concentration of 1 × 10^7^ CFU/mL for 24 hours at 30°C under agitation. At hourly intervals after inoculation, samples were collected from each strain, serially diluted, and spotted onto YPD agar plates for CFU enumeration. Regression analysis was performed to determine statistically significant differences in viability among strains. Three biological replicates were performed with two technical replicates.

### 2.4 Phospholipid quantification and evaluation

Lipid extraction and total inorganic phosphate (Pi) measurement was carried out using Mandala buffer as previously described and adapted to this study (Singh et al., 2011). Strains were cultured overnight in liquid YPD and then transferred to liquid MM for 15 hours. Strains were then washed with PBS and 5 × 10^8^ yeasts per sample were centrifuged and frozen in liquid nitrogen. Pellets were resuspended in 2 mL Mandala buffer (dH_2_O:ethanol:diethyl ether:pyridine:NH_4_OH (15:15:5:1:0.018; v/v)) and 50 mg of glass beads were added. Samples were vortexed for 30 seconds, sonicated three times for 30 seconds, vortexed again, and incubated in a 60°C water bath for 15 minutes. Following homogenization and centrifugation at 1,300 *× g* for 10 minutes at 4°C, the supernatant was transferred to a new tube and dried using SpeedVac. The pellet was resuspended in methanol and incubated at 37°C for 60 minutes, then centrifuged at 1,300 *× g* for 10 minutes at room temperature. The resulting supernatant was transferred to a new tube and 1 mL of chloroform and dH_2_O were added. They were then homogenized and centrifuged for 5 minutes to collect the lower organic phase containing the phospholipids.

For total phosphate (Pi) quantification, 120 μL of each lipid extract was digested with perchloric acid and incubated at 150°C for 2 hours. After cooling to room temperature, 900 μL of dH_2_O, 500 μL of 0.9% ammonium molybdate and 200 μL of 9% ascorbic acid were sequentially added with vortexing. Samples were incubated in water bath at 80°C for 30 minutes and absorbance was measured at 797 nm. A standard curve was constructed using NaH_2_PO_4_ (0-50 μM), and sample Pi concentrations were determined accordingly. Three biological replicates with nine technical replicates were analyzed.

Phosphatidylcholine quantification was performed using a commercial assay kit (MAK049, Sigma) in two biological replicates with three technical replicates. A qualitative evaluation of phospholipid profile was conducted using thin-layer chromatography (TLC). Silica gel plates pre-washed with chloroform:methanol:acetic acid:dH_2_O (60:50:1:4) eluent solution (Cartwright, 1993). After drying, 20 μL of wild-type sample was spotted onto the plate and mutant strains sample volume were normalized by total Pi quantification. After incubation in eluent solution, plates were developed using iodine vapor and plates were then photographed. Standards of PC (15:0-19:1-d7-PC, Avanti^®^), and PE (16:0-18:1-PE, kindly donated by Dr. Gabriel Silva Vignoli Muniz), were prepared at 10 mg/mL and 1 mg/mL, respectively, in chloroform:methanol (2:1). A quantitative analysis was conducted utilizing ImageJ v.1.53r to evaluate median pixel intensity (MPI) of the PC spot areas of each strain and results were normalized by wild-type PC spot area MPI. Six independent replicates were performed. Data were analyzed using one-way ANOVA with Dunnett’s correction, using the wild-type KN99α strain as the control.

### 2.5 Lipid droplet analysis

To evaluate lipid droplet formation, fungal strains were cultured overnight in YPD and then transferred to liquid MM for 15 hours at 30° C under agitation. After incubation, cells were washed with PBS and adjusted to a final concentration of 1 ×10^7^ CFU/mL. Lipid droplets were stained by incubating each sample with lipophilic dye Nile Red (Sigma) (Rostron and Lawrence, 2017) at a final concentration of 0.2 μM for 10 minutes at 30°C in the dark. Chitin content was assessed by staining with Calcofluor White (Fluorescent Brighetner 18, Sigma) at a final concentration of 100 μg/mL for 10 minutes. Mitochondrial activity was evaluated using MitoTracker™ Orange CMTMRos (ThermoFisher) at a final concentration of 0.2 μM under the same conditions. Stained and fixed cells were analyzed by both flow cytometry and fluorescence microscopy. For microscopy, samples were mounted on glass slides and imaged using a LEICA TCS SP5 confocal microscope equipped with a CorrL 405 63x/1.4-0.60 oil immersion objective. Fluorescence was detected across four excitation/emission channels (385–720 nm). For flow cytometry, stained cells were analyzed using BD VERSE flow cytometer equipped with PE-Texas Red (610/20 nm), Pacific-Blue (450/50 nm) and PE-A (586/15 nm) filters. Forward and side scatter parameters were used to gate single-cell populations and fluorescence intensity was recorded for at least 20,000 events per sample. Data were analyzed using FlowJo software (Treestar). Two biological replicates were carried out with two technical replicates. Statistical analyses were conducted using one-way ANOVA with Tukey’s correction for multiple comparisons.

### 2.6 Extracellular vesicles isolation

*C. neoformans* mutant strains (KN99α, *pct1Δ*, *opi3Δ*, and *opi3Δpct1Δ*) were cultured for extracellular vesicle (EV) isolation as described by Marina et al. (2020). Yeast cells in the exponential growth phase were first grown in YPD medium under agitation (150 rpm) for 48 hours at 30°C. Subsequently, cells were inoculated onto Sabouraud Dextrose Broth (SDB) plates at pH 5.5 to a final density of 4 × 10^7^ cells/mL and incubated for 24 hours at 30°C. After growth on SDB plates, cells were collected from the agar surface and resuspended in phosphate-buffered saline (PBS). The suspension was centrifuged at 5,000 × g for 15 minutes, and the supernatant was filtered through a 0.45 μm membrane and ultracentrifuged at 100,000 × g for 1 hour. The resulting pellet containing EVs was resuspended in 500 μL of PBS.

EV content was quantified indirectly by measuring ergosterol and protein concentrations using the Amplex Red Cholesterol Assay Kit (Thermo Fisher Scientific) for ergosterol and the Micro BCA Protein Assay Kit (Thermo Fisher Scientific) for protein, according to the manufacturer’s instructions. Vesicle size distribution and surface characteristics were analyzed by dynamic light scattering (DLS) using a Zetasizer instrument (Malvern Panalytical) to determine the hydrodynamic diameter of EVs. Data were analyzed using one-way ANOVA with Dunnett’s correction, using the wild-type KN99α strain as the control.

### 2.7 *In vitro* phagocytosis assay by murine macrophage

Phagocytosis assays were performed following a protocol adapted from Nicola and Casadevall (Nicola and Casadevall, 2012). Murine bone marrow-derived cells were cultivated in RPMI-1640 (Gibco) medium supplemented with 10% fetal bovine serum (FBS, Gibco), 20 ng/mL granulocyte-macrophage colony-stimulating factor (GM-CSF, ImmunoTools), and 50 μM β-mercaptoethanol (Sigma) for eight days. On the last day of differentiation, cells were recovered and the macrophages were counted and adjusted to 5 × 10^4^ cells/well, which were resuspended in RPMI-1640 + 10% FBS and transferred to a 96-well plate and incubated for 24 hours at 37°C in a 5% CO_2_ atmosphere.

Before macrophage infection, the wild-type and mutant strains were grown in liquid YPD for 24 hours. Cells were washed three times with PBS, counted, and resuspended in RPMI-1640. Fungal cells were opsonized in RPMI with 10 μg/mL of the anti-GXM monoclonal antibody 18B7 and inoculated into macrophage-containing wells at a multiplicity of infection (MOI) of 5. Plates were incubated for 2 hours and 24 hours at 37°C in a 5% CO_2_ atmosphere. After 2 hours of co-culture, wells were washed with PBS. In one plate, macrophages were lysed with 100 μL of distilled water. Lysates were diluted, plated on YPD agar, and incubated at 30°C for 48 hours to count colony-forming units (CFUs). In the second plate, 100 μL of RPMI 1640 + 10% FBS (with 500 IU of IFN-γ and 500 ng/mL LPS for the stimulated group) was added and incubated for an additional 24 hours. After incubation, macrophages were lysed, and CFUs were determined. The fungal viability was calculated using the formula: Viability (%) = {CFU (24h)/mean CFU (2h)} × 100. Data were analyzed using two-way ANOVA with Dunnett’s correction, using the wild-type KN99α strain as the control. All experiments included three technical replicates and were performed in three independent biological replicates.

### 2.8 Mitochondrial activity fluorimetric analysis

To investigate mitochondrial activity and cell reactive oxygen species (ROS) production, a fluorescence spectrophotometric analysis using Mitotracker™ Orange CMTMRos and 2’,7’-dichlorofluorescein diacetate, DCFDA (Sigma-Aldrich) stains, was carried out. The mutants and wild-type strains were grown in YPD and transferred to minimal medium for 15 hours as previously described. After that, yeast cells were washed with PBS, counted, and cell concentration was adjusted to 1 × 10^6^ yeast/mL in 500 μL for staining with either Mitotracker orange (0,2 μM) or DCFDA (10 μM) and incubated in the dark at 37°C for 30 minutes. Stained samples were then washed twice with PBS and 100 μL of each replicate was transferred to a 96 well plate for fluorimetric analysis in a Varioskan LUX (Thermo Fisher Scientific) plate reader. Mitotracker orange fluorescence was detected using 554/576 nm excitation/emission settings, while DCFDA was detected using 495/525 nm. Microscopic analysis of Mitotracker labeling was done using an EVOS M7000 Cell Imaging Systems (Invitrogen) microscope, coupled with Red Fluorescent Protein range emission (580 nm to 650 nm) LED light source. Microphotographs were taken with an exposure of 60 ms and 10.0 db gain. Each experiment was realized in technical triplicates and two biological replicates. One-Way ANOVA test with Dunnett correction was used for statistical analysis.

### 2.9 *G. mellonella* infection model assay

An *in vivo* virulence assay was conducted using *G. mellonella* larvae, following the protocol described by Mylonakis et al (Mylonakis et al., 2005). Larvae in their final development stage were weighed, and only those exceeding 200 mg were selected for the experiment. On the day of infection, *C. neoformans* cells previously grown for 15 hours in MM were resuspended in PBS, and the inoculum was adjusted to a final concentration of 5 × 10^6^ cells/mL. Each larva was injected with 10 μL of cell suspension into the last left proleg with a sterile Hamilton syringe. Following the injection, larvae were incubated at 37°C. For each fungal strain, 12 larvae were used. The negative control group was inoculated with 10 μL of PBS under the same conditions. Larval survival was monitored daily for 10 days. Data analysis was performed using the Mantel-Cox test.

### 2.10 *In vivo* murine infection model assay

The mice (*Mus musculus*), aged between 8 and 12 weeks and from the C57BL/6 strain, were housed under standard conditions at the animal facility of the Bi−Institutional Translational Medicine Platform/Fiocruz, with water and food provided *ad libitum.* All experimental procedures were approved by Ethics Committee of the University of São Paulo (Process No. 1305/2024) and conducted in accordance with institutional and national guidelines for animal care and use. Mice were infected via intratracheal route with 1 ×10^4^*C. neoformans* yeast cells previously cultured for 15 hours in liquid MM. For the survival curve analysis (n=5 per group), animals were monitored daily for clinical signs of morbidity. Mice exhibiting severe symptoms were euthanized according to the ethical recommendation. Briefly, mice were anesthetized with ketamine hydrochloride and xylazine hydrochloride (80–100 and 5–10 mg/kg, respectively), administered intramuscularly, followed by. euthanasia via intravenous administration of an overdose of the same anesthetic agents (ketamine hydrochloride, 240–300 mg/kg, and xylazine hydrochloride, 15–30 mg/kg). At 28 days post-infection, surviving animals were euthanized and visceral organs were exposed using sterile surgical instruments. The pulmonary lobes and brain tissue were collected to assess fungal burden. Samples were homogenized in PBS, and serial diluted, and plated on YPD plates for CFU counting. Data were analyzed using the Mantel-Cox test for survival growth curve and one-way ANOVA for CFU burden. Three independent experiments were realized.

### 2.11 Statistical analysis

All statistical analyses were performed using GraphPad Prism 8.3 (GraphPad Inc., San Diego, CA, USA). A p value < 0.05 was considered statistically significant.

## 3 Results

### 3.1 *Cn* Pct1 is essential for exogenous choline assimilation and GPC reestablishes growth of *opi3Δpct1Δ*

**Figure 2:**
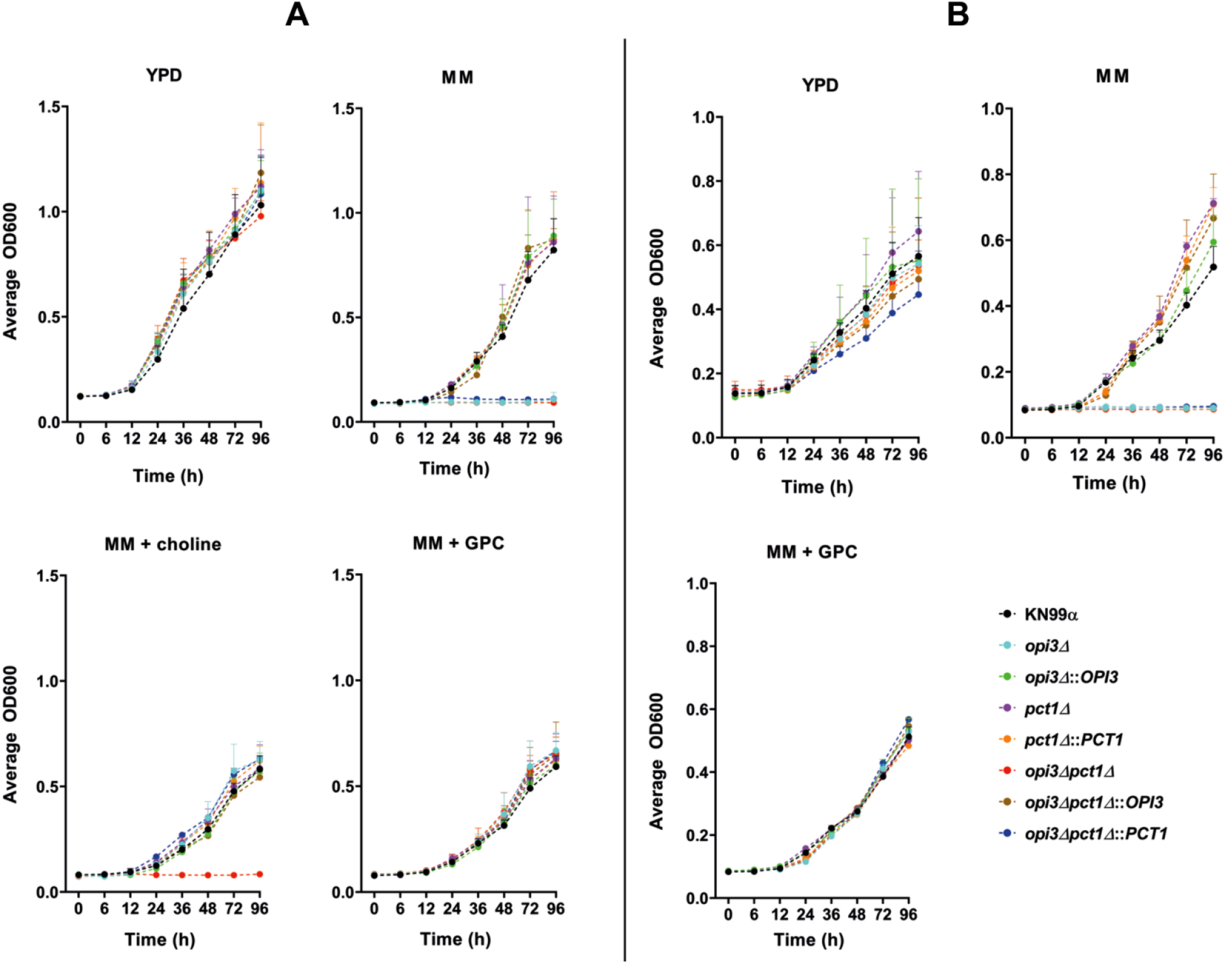
GPC reestablishes growth of the double mutant *opi3Δpct1Δ* in minimal medium. A total of 1 × 10^4^ cells from each strain were inoculated into different media for growth curve analysis, with optical density readings (600 nm) taken every 30 minutes over a period of 96 hours at **(A)** 30°C and **(B)** 37°C. The wild-type parental strain KN99α was used as control.

Deletion of *PCT1* appears to impair *C. neoformans’* ability to assimilate choline from the medium. This was evident from the impaired growth of the double mutant *opi3Δpct1Δ* in liquid MM supplemented with choline chloride (**Figure 2**). However, this mutant exhibited normal growth in YPD, similar to the wild-type, suggesting that it could acquire an alternative metabolite required for PC biosynthesis from rich medium. One likely candidate is the GPC pathway, a third route for PC production previously described in the literature (Głąb et al., 2016; King et al., 2024), potentially compensating for loss of *PCT1*. Supporting this, supplementation of liquid MM with only 10 μM GPC fully rescue the growth defect of *opi3Δpct1Δ* to the wild-type levels (**Figure 2**, **Supplementary Figure 3**). Interestingly, only high concentrations (500 μM) of PC (L-α-phosphatidylcholine type XVI-E) were sufficient to restore growth of the double mutant (**Supplementary Figure 3**). Furthermore, temperature did not affect growth of *OPI3* and or *PCT1* single mutants*;* however, GPC supplementation completely rescued growth defect of *opi3Δpct1Δ* observed in MM at both 30 and 37°C (**Figure 2**). These results indicate that while the Kennedy pathway is critical for choline assimilation in PC synthesis, the GPC reacylation pathway can compensate and plays a significant role in maintaining phospholipid synthesis in *C. neoformans*.

### 3.2 Loss of both *OPI3* and *PCT1* aggravates susceptibility to membrane stressors

**Figure 3:**
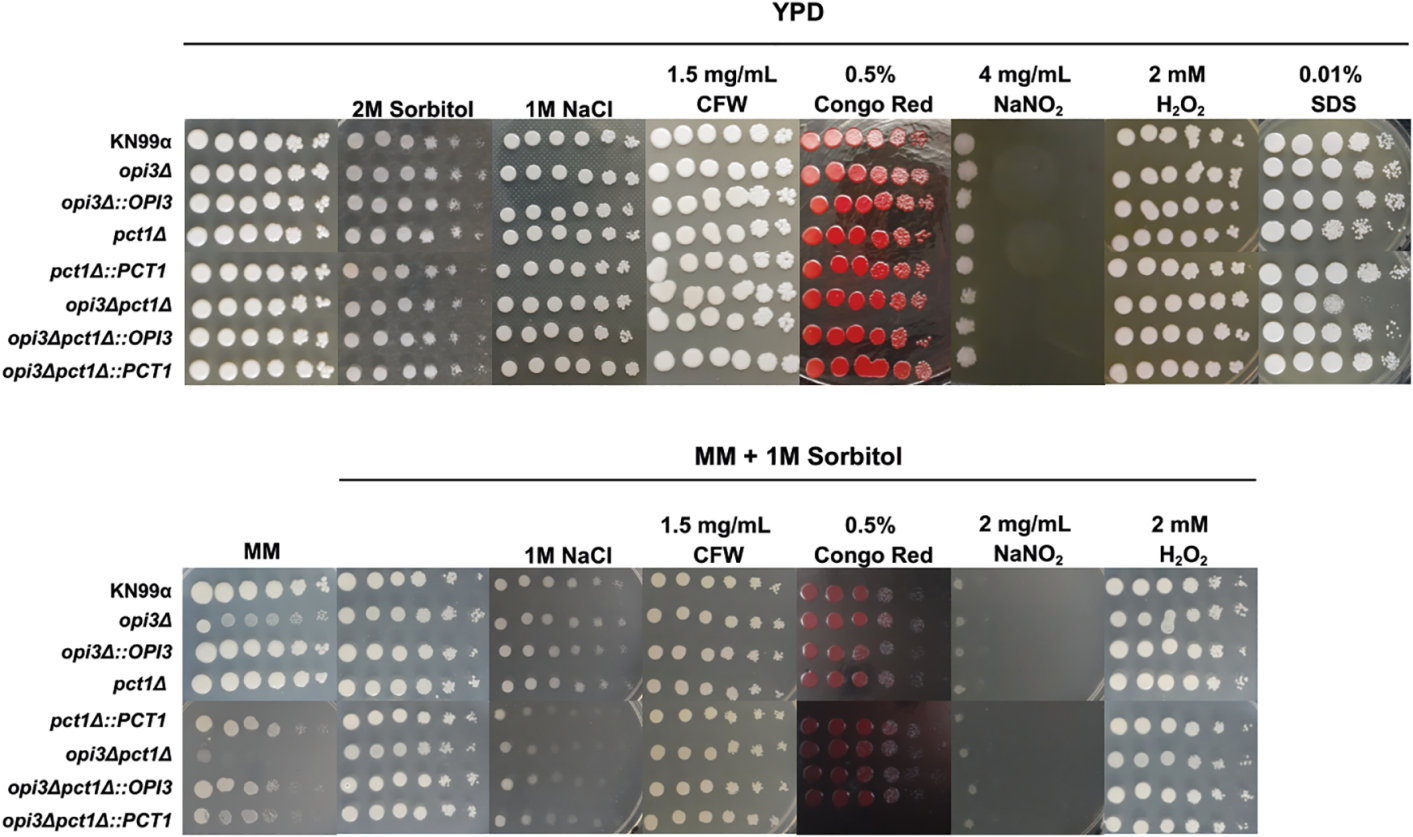
The double mutant *opi3Δpct1Δ* is more susceptible to membrane stress and cell wall. The mutants were inoculated in serial dilution (10^6^ – 10² cells) on YPD plates and MM plates supplemented with 1 M sorbitol containing different stressors agents and incubated at 30°C for 72 hours. The wild-type strain KN99α was used as control.

Lee et al. (2024) reported that sorbitol and polyethylene glycol (PEG) function as chemical chaperones, alleviating membrane-associated protein misfolding in *opi3Δ* mutant and rescuing growth under nutrient-limited condition (Lee et al., 2024). To determine whether a similar effect occurs in *opi3Δpct1Δ* mutant, growth was assessed in liquid MM supplemented with sorbitol. Sorbitol supplementation fully restored the growth defect of the double mutant (**Supplementary Figure 3**). To further investigate whether deletions of *OPI3* and *PCT1* compromises cell wall and plasma membrane integrity, strains were spotted onto MM agar plates containing 1M sorbitol and supplemented with stress-inducing agents. These included sodium dodecyl sulfate (SDS), a membrane-destabilizing detergent, and the cell wall-perturbing agents Calcofluor White and Congo Red, which bind to chitin polymers (Ram and Klis, 2006). The *opi3Δpct1Δ* double mutant showed pronounced growth defect on SDS-supplemented medium at 30°C (**Figure 3**) indicating alterations in membrane homeostasis. In contrast, the mutant exhibited wild-type growth on plates containing Calcofluor White and Congo Red despite reduction in cell wall chitin content after 15 hours under nutrient limited conditions, possibly indicating an active cell wall remodeling in response to phosphatidylcholine deprivation (**Supplementary Figure 4**). Single mutant strains did not exhibit any growth defects under these conditions.

To assess osmoregulatory capacity, all strains were grown on YPD and MM agar plates supplemented with either 1 M NaCl or 2M sorbitol. Under these conditions, no significant differences were observed between the mutants and the wild-type strain (**Figure 3**). Disruption of phospholipid metabolism has been reported to increase sensitivity to oxidative stress due to impaired mitochondrial function and respiration (Grant et al., 1997; Chang and Doering, 2018; Konarzewska et al., 2019). However, consistent with the osmostress assays, no growth defects were detected for either mutant or wild-type strains, when cultured on media supplemented with sodium nitrite (NaNO_2_) and hydrogen peroxide (H_2_O_2_) (**Figure 3**).

### 3.3 PC biosynthesis disruption impacts major *C. neoformans* virulence attributes

**Figure 4:**
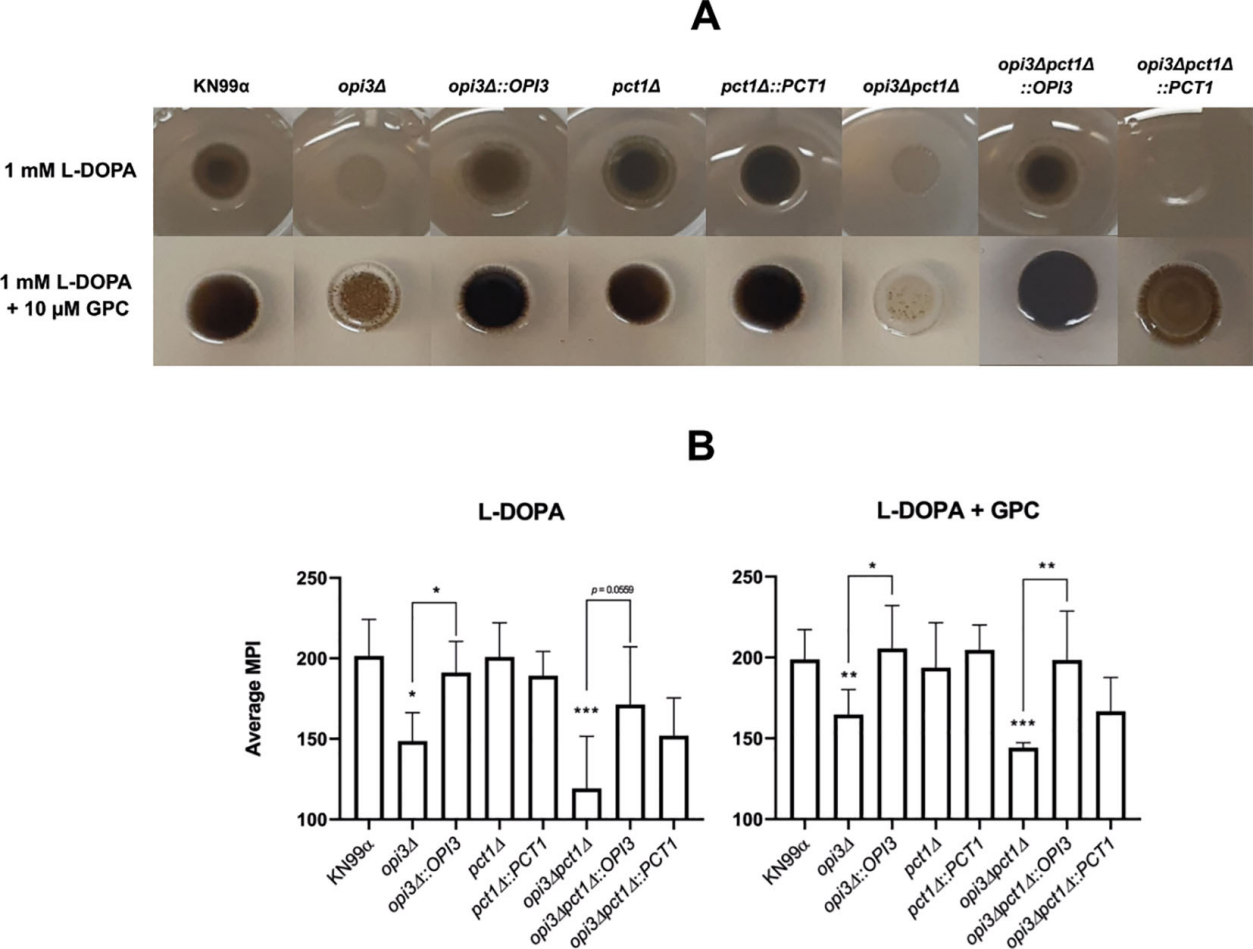
GPC rescues growth but not melanization of *OPI3-*deleted mutants at high temperature. **(A)** Mutant strains derived from the parental KN99α were grown on MM agar plates supplemented with 1 mM L-DOPA and incubated at 37°C to induce melanization. The medium was also supplemented or not with 1 mM choline chloride. Plates were photographed under identical lighting conditions at 96 hours post-inoculation. **(B)** Quantification of colony melanization was performed by pixel intensity analysis (MPI - Median Pixel Intensity) using ImageJ software. One-way ANOVA test with Tukey’s correction was used for statistical analysis. *p < 0.05; **p < 0.005; ***p < 0.001.

**Figure 5:**
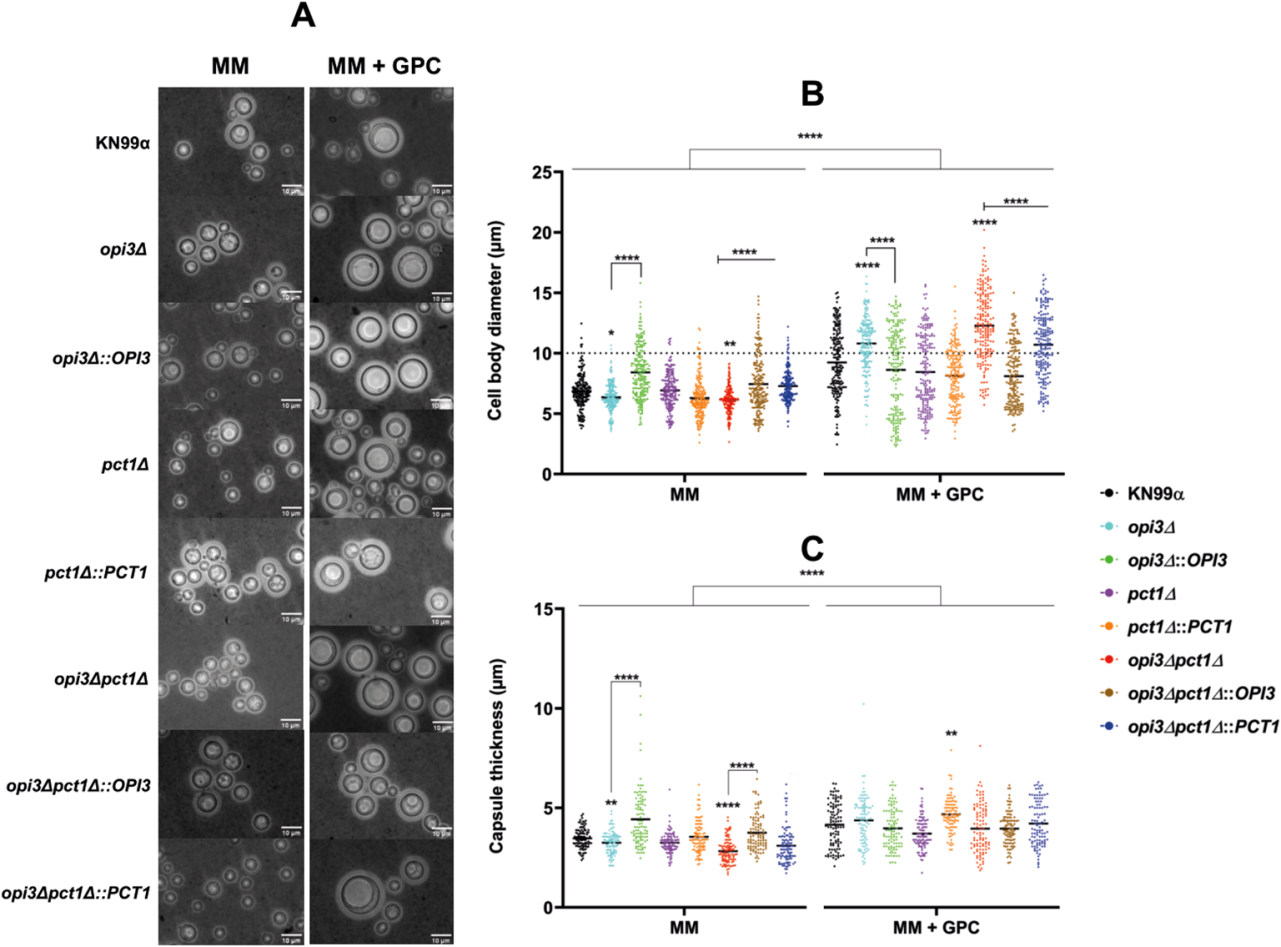
*OPI3*-deleted mutants exhibited reduced capsule size and titan cell formation in absence of GPC. Mutants derived from the parental strain KN99α were cultivated in liquid minimal medium (MM) supplemented or not with GPC at 30°C for 20 hours. The parental strain was used as a positive control in inducing medium. **(A)** Representative micrographs of cells stained with 20% India ink. Scale bar: 10 μm. **(B)** Measurement of cell body diameter (n= 200 cells) and **(C)** capsule thickness (n= 100 cells) after growth in liquid MM supplemented or not with 10 μM GPC. Capsule thickness was calculated as the difference between the total cell diameter and the cell body diameter. Cells above the dotted line represent the titan cell population (diameter >= 10 μm). Statistical analysis was performed using Kruskal Wallis test with Dunn’s correction for multiple comparisons and two-way ANOVA for comparison between treatments. *p < 0.05; **p < 0.005; ****p < 0.0001.

The ability to rapidly produce melanin is a critical *C. neoformans* virulence attribute, enabling the pathogen to survive inside host macrophage by protecting against reactive oxygen species (ROS), thereby favoring systemic dissemination (Wang and Casadevall, 1994; de Sousa et al., 2022). Melanin production was analyzed after 96 hours of induction at 37°C in MM agar plates supplemented with L-DOPA with or without GPC. Both *opi3Δ* and the *opi3Δpct1Δ* mutants exhibited reduced melanization, even in the presence of GPC (**Figure 4**). Additional virulence-related phenotypes, including polysaccharide capsule expansion and titan cell formation – key traits for immune evasion and lung infection establishment (Okagaki and Nielsen, 2012; Decote-Ricardo et al., 2019), were severely impaired under nutrient-limited conditions but fully restored upon GPC supplementation (**Figure 5**). Furthermore, GPC supplementation also increased the cell diameter of the opi*3Δpct1Δ* double mutant in comparison to the wild-type (**Figure 5B**), indicating physiological alterations linked to titanization. Other virulence-associated factors such as urease and phospholipase secretion were also investigated, but no differences were detected between mutants and wild-type strain (**Supplementary Figure 5**).

### 3.4 Cell viability and phospholipid composition is altered in the *opi3Δpct1Δ* double mutant after incubation under nutrient-limited medium

**Figure 6:**
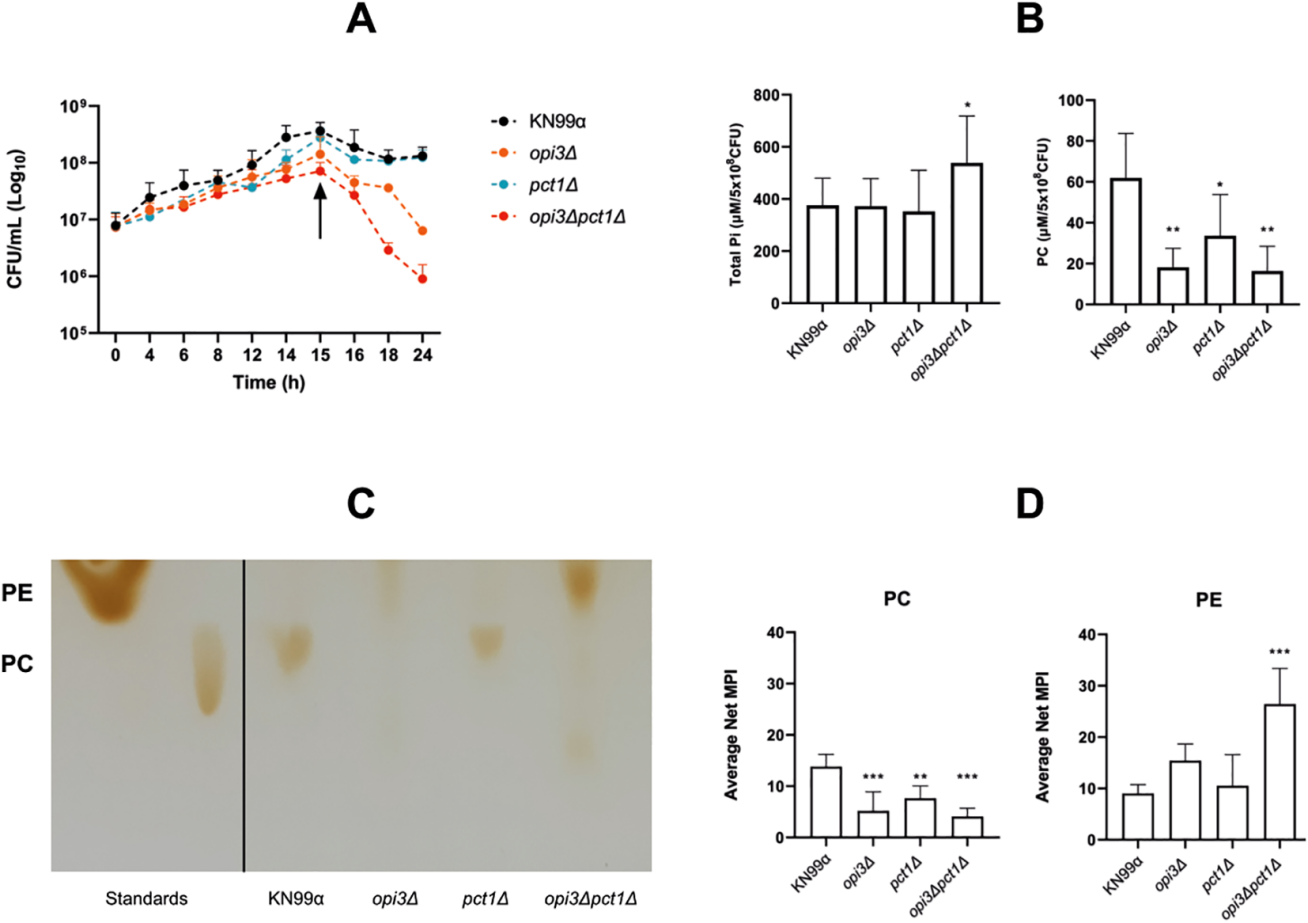
Disruption of PC synthesis reduces cell viability and alters lipid composition. **(A)** CFU of wild-type and mutant strains grown in liquid MM were measured every two hours after inoculation. The black arrow indicates the inflection point in the growth curves of *OPI3*-deleted mutants, followed by a decline in viability. **(B)** Phospholipids were extracted from cells grown for 15h under the same conditions for quantification of PC and total inorganic phosphate (Pi). PC levels were measured using the phosphatidylcholine assay kit (Sigma). For total Pi quantification, extracted lipids were digested with perchloric acid at 150°C and homogenized in dH_2_O, 0.9% ammonium molybdate and 9% ascorbic acid, prior to absorbance reading. Results were normalized to 5 × 10^8^ CFU **(C)** Thin layer chromatography (TLC) utilizing chloroform:methanol:acetic acid:H_2_Od (60:50:1:4) eluent system. After drying, 20 μL of wild-type sample was spotted onto the plate and mutant strains sample volume were normalized by total Pi quantification. Iodine vapor was used to develop plates that were photographed in same lighting conditions. **(D)** Average net median pixel intensity (MPI) of PC and PE TLC spots were analyzed utilizing ImageJ software. Statistical analysis was performed using one-way ANOVA with Dunnett’s correction for multiple comparisons. *p < 0.05; **p < 0.005; ***p < 0.001.

Cell viability of PC mutants was accessed in MM by culturing cells and determining CFUs over time. The results indicated that although the *opi3Δpct1Δ* mutant was capable of growth, its growth rate was markedly slower than the wild-type, with a notable decline in viability occurring around 15 hours post-inoculation. In contrast, *opi3Δ* mutant maintained viability for a longer period before exhibiting a gradual reduction (**Figure 6A**). These observations guided the design of subsequent experiments, including PC quantification, using viable cells in a PC-depleted state. Under these conditions, wild-type, *opi3Δ*, *pct1Δ* and *opi3Δpct1Δ* strains were cultivated and their phospholipids were extracted for analysis. Quantification of total Pi and PC levels revealed a significant reduction in PC content in the *opi3Δ* and *opi3Δpct1Δ* mutants, and to a lesser extent in the Kennedy pathway mutant, *pct1Δ*; however, total Pi levels were increased only in the *opi3Δpct1Δ* mutant, suggesting accumulation of other phospholipids species (**Figure 6B**). Chromatographic and quantitative analysis showed that loss of Opi3 was the major determinant of PC depletion in *C. neoformans*, while *PCT1* appears to play a constitutive role in PC biosynthesis beyond its function in the salvage pathway (**Figures 6C,D**). Notably, deletion of *PCT1* resulted in elevation of PE levels in the *opi3Δpct1Δ* mutant (**Figures 6C, D**), reflecting a compensatory mechanism to preserve membrane homeostasis under PC-depletion condition.

### 3.5 Loss of both *OPI3* and *PCT1* induces rapid neutral lipid accumulation

**Figure 7:**
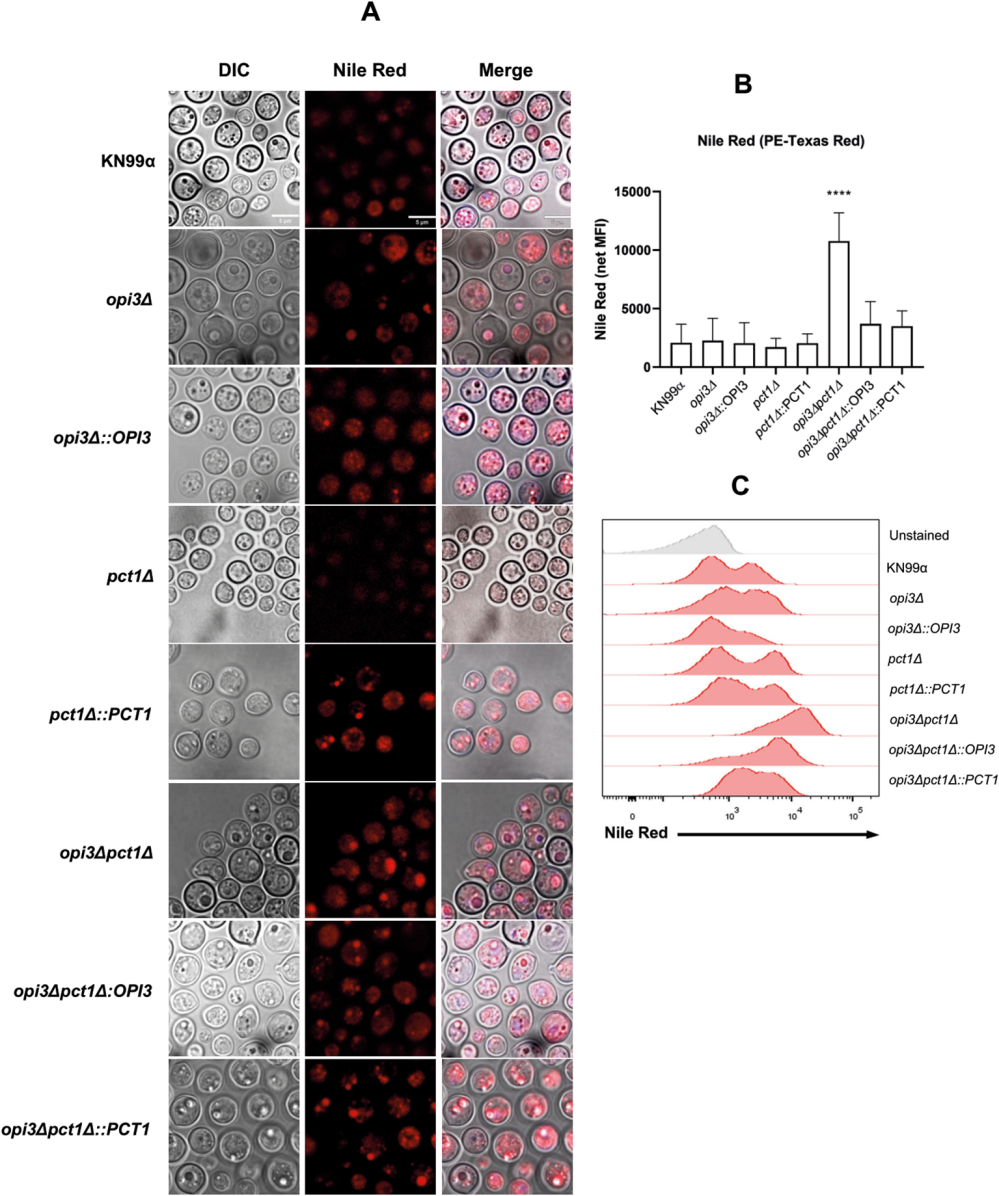
The *opi3Δpct1Δ* mutant rapidly accumulates neutral lipids. Fluorescence microscopic and flow cytometric analysis using Nile Red staining were performed on PC biosynthesis pathways mutant strains grown in liquid MM for 16 hours. **(A)** Representative fluorescence micrographs of Nile Red-stained cells captured at 63×magnification using an emission filter in 500 to 700 nm range. Scale bar represents 5 μm. **(B)** Average mean fluorescence intensity (MFI) of 20,000 stained cells normalized to unstained control measured using the PE-Texas Red filter. **(C)** Histogram showing red-shift fluorescent emission profiles of the samples. One-way ANOVA with Tukey’s correction for multiple comparisons was used for statistical analysis. ****p < 0.0001.

Previous studies with yeasts have shown that deletion of *OPI3* gene disrupts lipid homeostasis and leads to increased lipid droplet size (Lee et al., 2024). Lipid droplets are cellular organelles composed of a phospholipid monolayer surrounding a neutral lipid core rich in triacyclglycerols (TAGs) and sterols (Vevea et al., 2015). They play roles in various physiological functions such as lipid storage, membrane homeostasis, stress responses and autophagy (Graef, 2018). To investigate potential alterations in lipid metabolism, PC synthesis mutant strains were cultured in liquid MM for 15 hours and analyzed by flow cytometry and fluorescence microscopy using Nile Red staining. The results revealed that the *opi3Δpct1Δ* strain displayed a mean fluorescence index (MFI) approximately twice that of the wild-type, which indicates a significant accumulation of neutral lipids. Reintroduction of either *OPI3* or *PCT1*, significantly reduced the Nile Red fluorescence (**Figure 7**). Notably, the *opi3Δ* mutant did not show a significant increase in lipid accumulation, possibly due to compensation by the intact Kennedy pathway utilizing the TAG precursor, diacylglycerol, for PC biosynthesis.

### 3.6 Loss of *OPI3* and *PCT1* increases cellular ROS levels

**Figure 8:**
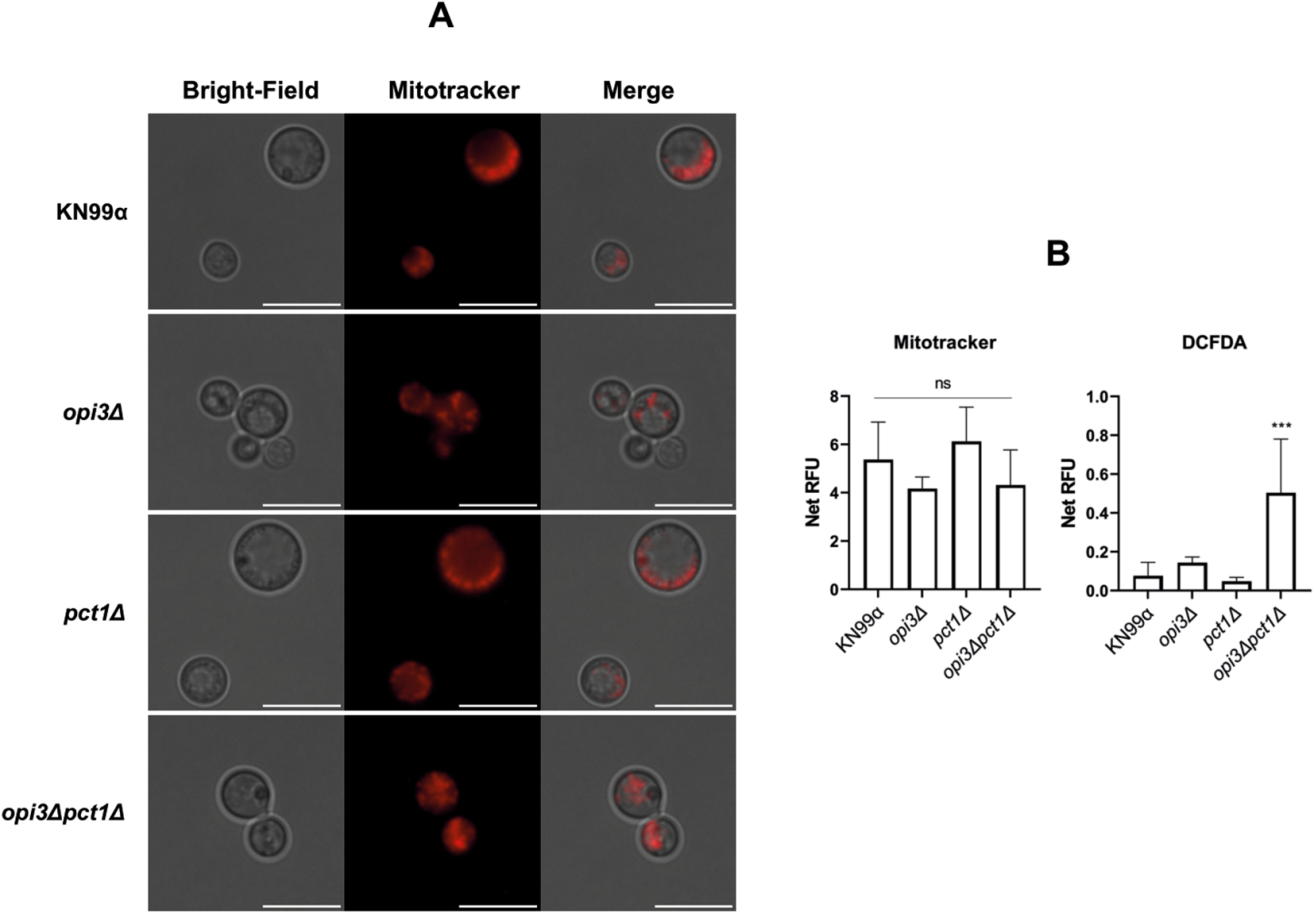
Microscopic and fluorimetric analysis of mutant and wild-type strains stained with Mitotracker orange and DCFDA. Phosphatidylcholine synthesis mutant and wild-type strains were grown in YPD and transferred to minimal medium for 15 hours for nutrient depletion before staining with 200 nM Mitotracker orange for mitochondrial activity analysis or 10 μM DCFDA for cell ROS production evaluation. **(A)** Microscopic photography utilizing Mitotracker orange probe. KN99 wild-type strain was used as control. White bar represents 10 μm. **(B)** Fluorescence from samples stained with Mitotracker orange and DCFDA were measured using a Varioskan LUX microplate reader. RFU stands for Relative Fluorescence Unit and it was obtained after subtracting stained fluorescence values from unstained control sample values. The results represent the average between two biological replicates containing three technical replicates. One-Way ANOVA test with Dunnett correction was used for statistical analysis. ***p < 0.001.

Previous studies have shown that PC depletion in *S. cerevisiae* yeast cells negatively impacts mitochondrial morphology and function (Bao et al., 2021). To evaluate mitochondrial activity and ROS levels in the disrupted PC synthesis mutant strains, Mitotracker orange and DCFDA staining fluorimetric analysis were carried out (**Supplementary Figure 5**, **Figure 8**). Mitotracker is a membrane potential-dependent probe that labels mitochondria in live cells, while DCFDA is used to detect cellular ROS and H_2_O_2_ (Maghzal et al., 2012). Our study observed no impact in mitochondrial activity after 15 hours under nutrient limited conditions (**Figure 8**), however, intracellular levels of ROS were elevated in the *opi3Δpct1Δ* double mutant (**Figure 8B**). Although endoplasmic reticulum stress and fatty acid degradation are known to increase cellular ROS levels (Morano et al., 2012), occurrence of mitochondrial defects should not be discarded even though our results pointed to no alterations in membrane potential.

### 3.7 Deletion of key components of phosphatidylcholine synthesis alters extracellular vesicles size distribution

Analysis of EV size distributions revealed distinct profiles among the mutants compared to the wild-type (KN99α). The wild-type strain exhibited a narrow size distribution, with vesicles predominantly around 100–150 nm in diameter. The *pct1Δ* mutant displayed a slightly broader distribution, with vesicles mainly between 100–200 nm, while the *opi3Δ* mutant showed a broader range, from approximately 100–300 nm. The *opi3Δpct1Δ* double mutant exhibited the widest distribution, ranging from 100 up to 400 nm, indicating an additive effect of the two mutations on vesicle heterogeneity (**Supplementary Figure 6A**). Despite these differences in vesicle size, total protein (**Supplementary Figure 6B**) and sterol content (**Supplementary Figure 6C**) per 10^9^ yeast cells were not significantly different among the strains, confirming that the observed size changes were not due to variations in overall vesicle yield.

### 3.8 The double deletion of *OPI3* and *PCT1* affects fungal survival after phagocytosis and impairs virulence *in vivo*

**Figure 9:**
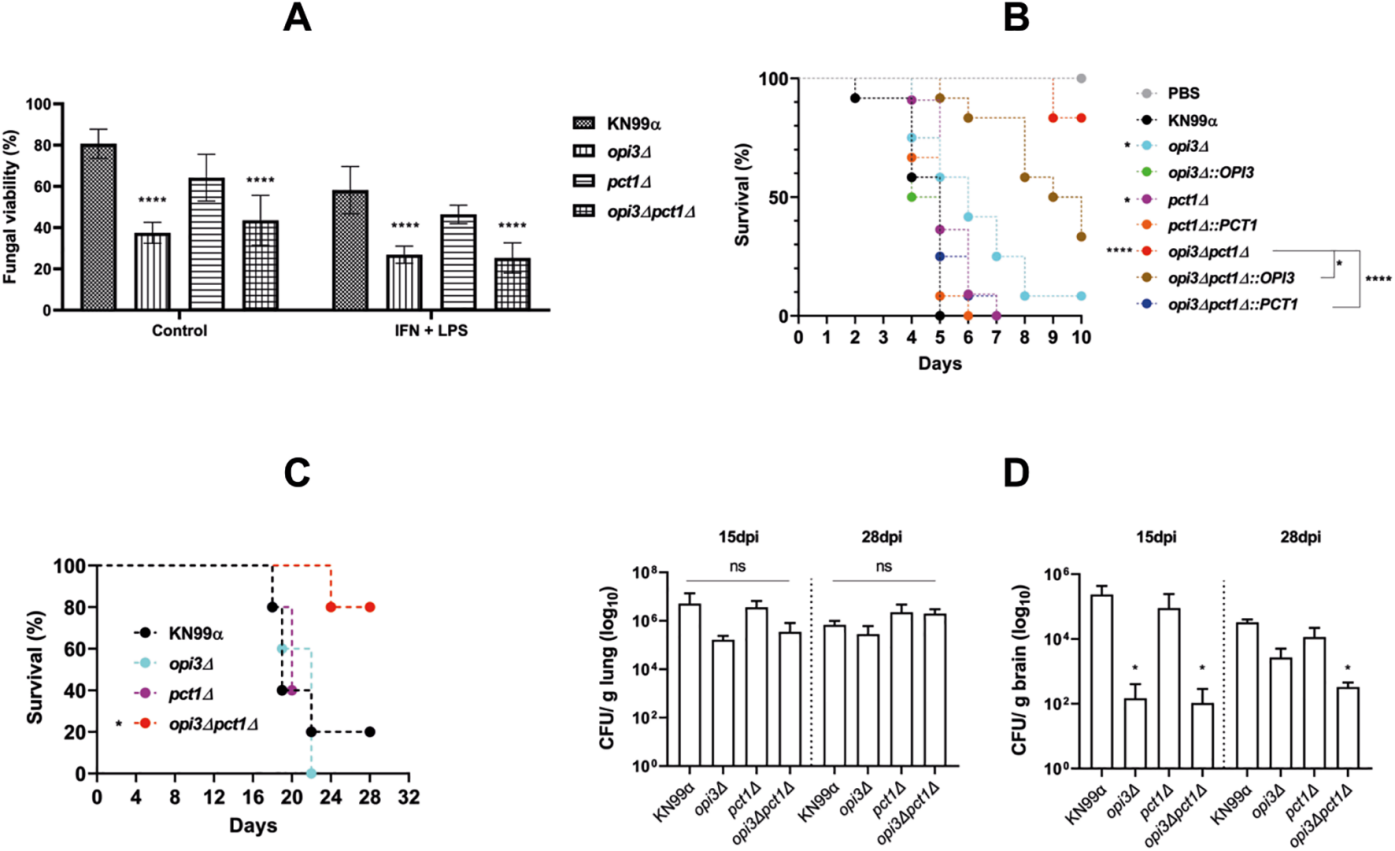
The *opi3Δpct1Δ* double mutant exhibited reduced survival after murine macrophages phagocytosis and impaired virulence in animal models of *C. neoformans* infection. **(A)** Murine macrophages were co-culture with YPD grown yeast cells for 2 h and 24 (h). After each time point, macrophages were lysed and yeast CFUs were determined by plating on YPD. Yeast survival (%) was calculated as the ratio of CFU at 24h to CFU at 2h × 100. Statistical analysis was performed using two-way ANOVA with multiple comparison. **(B)***G*. *mellonella* survival curve following infection with mutant strains. Yeast cells were grown for 15 hours in liquid MM, washed, and resuspended in PBS. A total of 10 µL of PBS containing 5 × 10^4^ cells were injected in the larvae (n=12 per group. The parental strain KN99α served as a positive control, PBS as the negative control. Statistical analysis was performed with Mantel-Cox test. **(C)** Survival curve and fungal burden of the lungs and brain from infected mice. Yeast cells were grown in liquid MM for 15 hours and inoculated via intratracheal route. Mice showing signs of morbidity were euthanized and **(D)** organs were collected after 15 and 28 days post-infection for CFU determination. Survival analysis was performed using Mantel-Cox test, while fungal burden was analyzed by one-way ANOVA with Dunnett’s correction for multiple comparisons. *p < 0.05; ****p < 0.0001.

To evaluate virulence-related phenotypes, the survival and proliferation of *opi3Δ, pct1Δ* and *opi3Δpct1Δ* mutant strains were first investigated after co-culture with murine macrophages. Following phagocytosis, the killing rate of *OPI3* deleted mutants (*opi3Δ* and *opi3Δpct1Δ*) was significantly higher compared to the *pct1Δ* mutant and wild-type strains (**Figure 9A**). However, *in vivo* assays using *G. mellonella* larvae revealed that the double mutant was hypovirulent, while the *opi3Δ* and *pct1Δ* single mutant were slightly less virulent compared to the wild-type strain (**Figure 9B**). To determine whether these results would be translated to a mammalian host, mice were infected with the mutant strains and survival curves along with fungal burdens were analyzed. The *opi3Δpct1Δ* strain was confirmed to be hypovirulent, as infected mice survived longer than those infected with wild-type, *opi3Δ*, and *pct1Δ* strains (**Figure 9C**). Interestingly, fungal burden revealed that although the double mutant colonized the lungs at similar levels to the wild-type, its presence in the brain was markedly reduced. These findings suggest that while *opi3Δpct1Δ* mutant can establish pulmonary infection, its ability to disseminate and/or survive in central nervous system is virtually ablated, resulting in reduced overall pathogenicity.

## 4 Discussion

The biosynthesis of PC is essential for maintaining cellular homeostasis and virulence in fungi. The *de novo* pathway has been extensively characterized across fungal systems, where it plays a crucial role in PC synthesis and membrane integrity. In *S. cerevisiae*, deletion of *OPI3* activates endoplasmic reticulum (ER) - mediated unfolded protein response (UPR), impairs growth under choline-limiting conditions, and alters PC acyl-chain composition through enhanced choline recycling (Carman and Henry, 1999; Boumann et al., 2006; Hancock et al., 2006). Similarly, in *Aspergillus fumigatus*, deletion of *choC* disrupts lipid composition, compromising viability, morphology and cell wall integrity, ultimately reducing virulence (Pan et al., 2024). In contrast, in *C. albicans*, deletion of the orthologous PE methyltransferases PEMT1 and PEMT2 increases virulence via accumulation of PE synthesized through the Kennedy pathway (Tams et al., 2019). In *C. neoformans*, deletion of *OPI3* causes choline auxotrophy, lipid imbalance, and reduced capsule formation but does not abolish infectivity, indicating that host-derived choline can sustain virulence (Lee et al., 2024).

The Kennedy pathway conserved from fungi to mammals synthesizes PE and PC via incorporation of free ethanolamine and choline (Choi et al., 2004; Gibellini and Smith, 2010; McMaster, 2018). Its contribution to fungal pathobiology is variable. In *Metarhizium robertsii* deletion of *MoPCT1*, does not affect growth, morphology, or appressorium formation, but reduces virulence in a larval infection model (Chen et al., 2018). In *Magnaporthe oryzae* loss of *PCT1*, compromises appressorium turgor, diminishing host penetration and pathogenicity (Xu et al., 2023). By contrast, *F. graminearum* mutants defective in the Kennedy pathway display altered colony morphology and conidiation but retain wild-type virulence in wheat (Wang et al., 2019). In *C. neoformans*, Kennedy pathway is active but cannot compensate for the deletion of *CHO1* (Konarzewska et al., 2019). In accordance, our results show that loss of *PCT1* alone does not affect virulence or key phenotypic traits in *C. neoformans*, highlighting both interspecific, and potentially intraspecific, variability in the physiological role of Kennedy pathway in fungi.

The simultaneous disruption of the *de novo* and Kennedy pathways has not been previously characterized in pathogenic fungi. Our findings demonstrate that concurrent deletion of *OPI3* and *PCT1* compromises growth, membrane integrity, and pathogenicity in *C. neoformans*. The interplay between the *de novo* and Kennedy pathways underscores the importance of metabolic redundancy for fungal survival. Whereas deletion of *OPI3* alone resulted in relatively mild phenotypes, additional loss of *PCT1* markedly exacerbated growth defects and stress sensitivity, demonstrating their complementary roles in sustaining PC biosynthesis. Similar variability has been reported in other fungi, including *Candida albicans* and *Fusarium graminearum*, where disruption of PC biosynthetic pathways affects lipid composition, colony morphology, and virulence in species-specific manners (Davis et al., 2018; Wang et al., 2019). In *C. neoformans*, the additive effects observed in the double mutant suggest that both pathways contribute to PC production under standard and nutrient-limited conditions. The absence of a canonical CDP-choline phosphotransferase in *C. neoformans* supports the hypothesis that a substrate-ambiguous enzyme, such as Ept1p, may provide functional overlap between pathways. Biochemical characterization of Ept1p and its substrate specificity will be essential to define the extent of this compensatory mechanism.

At the mechanistic level, PC depletion in yeast is known to induce PE/PC asymmetry, altering membrane curvature and rigidity and generating stored curvature elastic (SCE) stress that activates the UPR (Thibault et al., 2012). In *S. cerevisiae*, Pct1 senses such membrane stress by recognizing elevated PE and diacylglycerol (DAG) levels and initiating CDP-choline synthesis (Haider et al., 2018). In *C. neoformans*, deletion of *OPI3* induces expression of *IRE1* and *HXL1* under choline starvation, culminating in UPR pathway activation (Lee et al., 2024), and similar lipid-driven ER stress has been reported in *Aspergillus fumigatus* (Pan et al., 2024). The *C. neoformans*, *opi3Δpct1Δ* mutant failure to restore PC levels through Pct1-mediated CDP-choline synthesis to alleviate SCE stress correlates with increased intracellular ROS, accumulation of PE and fatty acyl chains in lipid droplets, and sustained membrane stress. Perturbation of DAG levels and membrane composition may also affect protein kinase C-dependent cell wall integrity signaling, as well as cAMP/PKA and Ras pathways that regulate morphogenesis and stress adaptation (Morano et al., 2012).

Disruption of PC biosynthesis also had profound consequences for virulence-associated traits in *C. neoformans*. The *opi3Δpct1Δ* mutant exhibited reduced capsule formation, impaired titan cell development, and diminished melanization, key attributes required for immune evasion and dissemination (Okagaki and Nielsen, 2012; Decote-Ricardo et al., 2019; de Sousa et al., 2022). Supplementation with L-α-glycerophosphorylcholine (GPC), but not choline, restored capsule and titan cell phenotypes, implicating GPC-derived reacylation in maintaining membrane lipid homeostasis required for fungal morphogenesis and virulence. In contrast, melanization defects persisted despite GPC supplementation, indicating a greater dependence on intact membrane organization and vesicle-mediated secretion than on restoration of total PC levels. In *C. neoformans*, melanin synthesis and deposition depend on endomembrane and extracellular vesicles that deliver laccase and melanin intermediates to the cell wall (Rodrigues et al., 2008; Eisenman et al., 2009). Accordingly, extracellular vesicles isolated from the double mutant exhibited altered size distributions, indicative of defects in vesicle biogenesis or trafficking. Such alterations likely also contribute to capsule and cell wall abnormalities by impairing the delivery of capsule polysaccharides, and key cell wall-synthesizing enzymes, including chitin synthases and β-1,3-glucan synthase (Fks1), to the plasma membrane (Cabib et al., 2001). Supporting this model, mammalian cells harboring thermosensitive choline phosphate cytidylyltransferase exhibit selective post-Golgi trafficking defects under PC-limiting conditions, despite preserved ER-to-Golgi transport and Golgi morphology (Testerink et al., 2009; Sarri et al., 2011).

Remarkably, GPC or osmotic stabilizers fully restored growth and viability of the double mutant, supporting the existence of a compensatory reacylation pathway that bypasses the canonical PC pathways. GPC-derived reacylation is conserved in fungi and plants but is absent in mammals (Głąb et al., 2016; Anaokar et al., 2019; King et al., 2024). Although not yet functionally characterized in *C. neoformans*, putative orthologs of key the enzymes lysophospholipid acyltransferase 1 (CNAG_00365) and glycerophosphocholine acyltransferase 1 (CNAG_03991) were identified in the genome. Functional characterization of theses enzymes will be critical to establish whether GPC reacylation sustains PC levels independent of *de novo* and Kennedy pathways. Those experiments will provide evidence to understand the metabolic plasticity that enables *C. neoformans* to adapt to nutrient-limited host environments.

Although deletion of *OPI3* alone is not sufficient to abolish virulence in a murine model, as also reported by Lee et al. (2024), concomitant deletion of *PCT1* significantly attenuated *C. neoformans* pathogenicity, revealing a critical role for coordinated phosphatidylcholine (PC) biosynthesis during infection. Intriguingly, despite pulmonary fungal burdens comparable to the wild-type strain, the *opi3Δpct1Δ* double mutant exhibited a reduced fungal burden in the brain after 28 days post-infection, indicating a defect in dissemination and/or survival within the CNS. This phenotype likely reflects additive, niche-specific constraints on PC metabolism. Limited availability of GPC in the CNS (Himmelreich et al., 2001), combined with loss of melanin and impaired vesicle-mediated processes, and may restrict proliferation within brain tissue. In parallel, dissemination itself may be compromised, as intravascular proliferation is a prerequisite for brain invasion (Wang et al., 2025), and the double mutant displayed delayed growth in serum, suggesting reduced fitness during hematogenous spread. Dissemination may be further hindered by increased cell size, potentially driven by elevated GPC levels within pulmonary cryptococcomas (Himmelreich et al., 2003), and by enrichment of titan cells which are known to impede traversal of biological barriers, including the blood-brain barrier (Okagaki and Nielsen, 2012; Reuwsaat et al., 2021; Denham et al., 2022).

Together, these findings support a model in which *Cryptococcus* pulmonary colonization tolerates substantial flexibility in PC biosynthetic routes, whereas efficient CNS invasion requires coordinated activity of the *de novo* CDP-DAG-dependent methylation and Kennedy pathway. Consistent with this view, our lipid quantification indicate predominant reliance on the CDP-DAG pathway in the wild-type strain, with the Kennedy and reacylation pathways providing compensatory capacity when individual routes are disrupted. However, reliance on reacylation alone appears insufficient to sustain growth during systemic fungal dissemination. Supporting this idea, transcriptomic analyses of clinical isolates recovered from human subarachnoid space and within cerebrospinal fluid reveal simultaneous expression of genes from all PC biosynthetic pathways during infection (Yu et al., 2021). These findings point to niche-specific requirements for PC biosynthesis during infection, underscoring metabolic flexibility as a core virulence strategy.

Collectively, our results establish that PC metabolism integrates lipid homeostasis, morphogenesis, and pathogenicity in *C. neoformans*. The apparent redundancy among the *de novo*, Kennedy, and GPC reacylation pathways ensures metabolic resilience but also reveals potential vulnerabilities. Because GPC reacylation is absent in mammals, its enzymatic machinery emerges as a promising antifungal target. Future screening of acyltransferase inhibitors and structure-function studies of the corresponding enzymes may yield novel therapeutic strategies that selectively collapse PC biosynthesis in pathogenic fungi (Sant et al., 2016; Pan et al., 2018).

## 5 Conclusion and perspectives

This work identifies phosphatidylcholine biosynthesis as a key determinant of *C. neoformans* physiology and virulence. The combined disruption of *OPI3* and *PCT1* destabilizes membrane integrity, alters lipid composition, and compromises critical virulence traits, ultimately impairing dissemination to the central nervous system. The capacity of GPC-derived reacylation to restore viability underscores its importance as a metabolic safeguard under stress conditions. Integration of lipidomics, transcriptomics, and vesicle proteomics will be instrumental to unravel how lipid remodeling intersects with morphogenesis and host adaptation. Functional validation of GPC acyltransferases and exploration of their inhibition as antifungal strategies represent exciting avenues for future research. By elucidating the interplay between membrane metabolism and pathogenic behavior, this study provides a foundation for targeting metabolic plasticity in fungal pathogens.

## Data Availability

The original contributions presented in the study are included in the article/**Supplementary Material**. Further inquiries can be directed to the corresponding author.
