# Supplementary material for: Phosphatidylcholine biosynthesis pathways in Cryptococcus neoformans: functional interplay and impact on virulence
Source: Front Cell Infect Microbiol. 2026 Jan 27;15:1736171. doi: 10.3389/fcimb.2025.1736171 (PMC12886497; doi:10.3389/fcimb.2025.1736171)
Supplement: Supplementary file 1 [file DataSheet1.docx]

**Timboni, FS. Supplementary material**


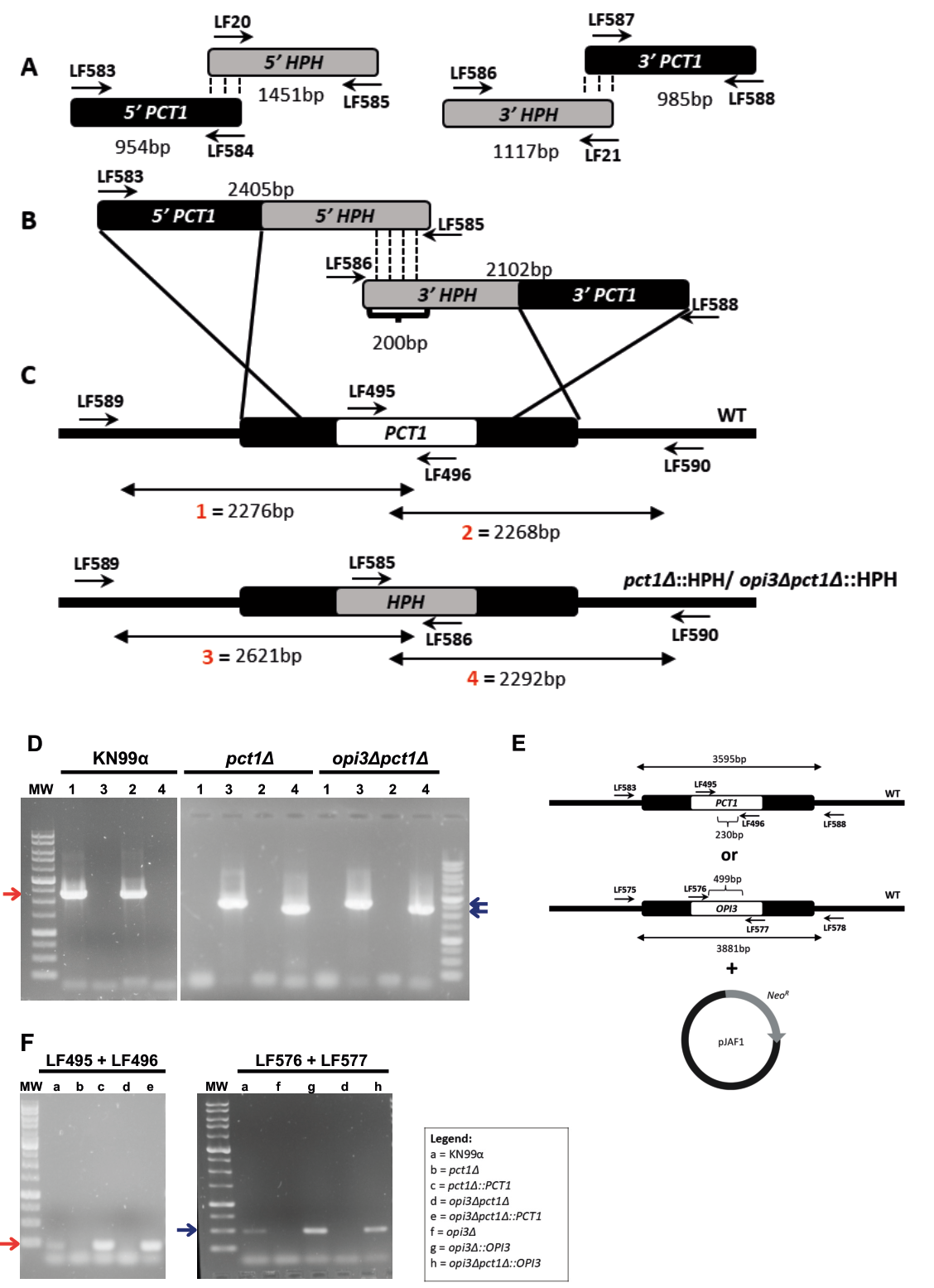


**Supplementary Figure 1. Procedure for gene deletion and reconstitution in *C. neoformans* using fragments of the HYG and NEO selection markers.** (A) First, the 5’ and 3’ flanking regions of the target gene were amplified by PCR using primers LF583 and LF584, and LF587 and LF588, respectively. The 5’ and 3’ fragments of the HYG marker were also amplified using primers LF20 and LF585, and LF586 and 6 LF21, respectively. In the second step (B), the 5’ flanking region fragment and the 5’ fragment of the HYG marker were fused and amplified by PCR using primers LF583 and LF585 (Double Joint PCR). The same procedure was applied to the 3’ fragments using primers LF586 and LF588. (C) The two resulting fragments were combined and delivered into the cell nucleus via biolistic transformation, where three homologous recombination events replaced the target gene with the cassette containing the *HPH* selection marker. (D) Electrophoresis analysis in agarose gel (0.7%) confirming deletion of PCT1 in the *pct1Δ* and *opi3Δpct1Δ* constructed mutants. Red arrow show amplification of target gene fragments and blue arrows show amplification of cassette deletion fragments. (E) Mutant reconstitution was performed by biolistic co-transformation using amplified *PCT1* (LF589 and LF590) or *OPI3* (LF575 and LF578) locus and pJAF1 plasmid containing neomycin resistance cassette (*Neo^R^*). Amplification of internal fragments of *PCT1* (LF495 and LF496) and *OPI3* (LF576 and 577) confirmed reconstitution. (F) Electrophoresis analysis in agarose gel (1.2%) confirming deletion and reconstitution of OPI3 and PCT1 transformants. Red arrow show amplification of PCT1 internal gene fragment (230 bp) and blue arrow show amplification of OPI3 internal gene fragment (499 bp). ***OPI3***, gene encoding phosphatidylethanolamine methyltransferase. ***PCT1***, gene encoding choline cytidyltransferase; ***HPH***, gene conferring resistance to Hygromycin B;


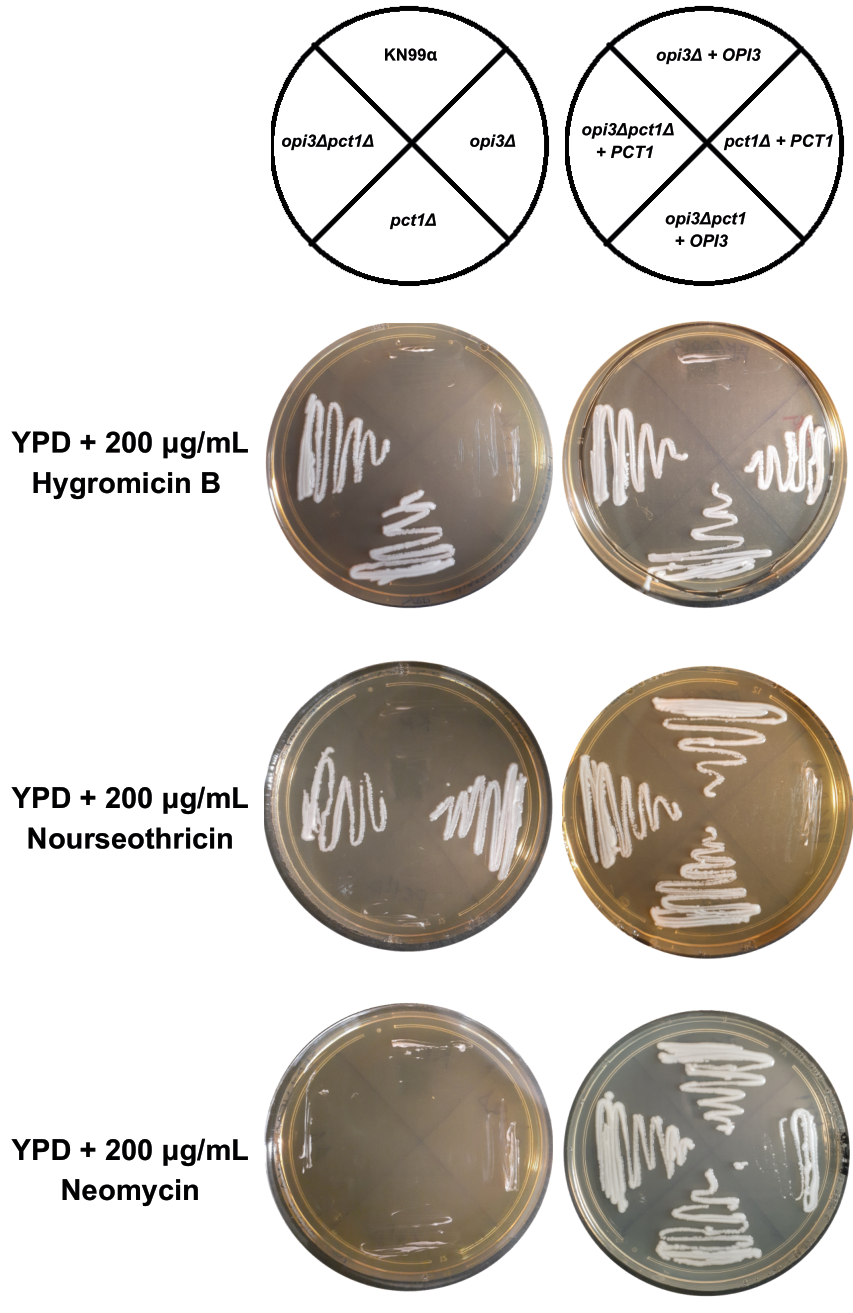


**Supplementary Figure 2. Wild type KN99α, mutant and reconstituted strains grown in different selection marker medium.** Strains were grown for 72 h in YPD supplemented with: hygromycin B for selection of constructed *pct1Δ* and *opi3Δpct1Δ* mutants; nourseothricin for selection of *opi3Δ* and *opi3Δpct1Δ* mutants; neomycin for selection of reconstituted strains *opi3Δ*::*OPI3*, *pct1Δ*::*PCT1*, *opi3Δpct1Δ*::*OPI3* and *opi3Δpct1Δ*::*PCT1*.


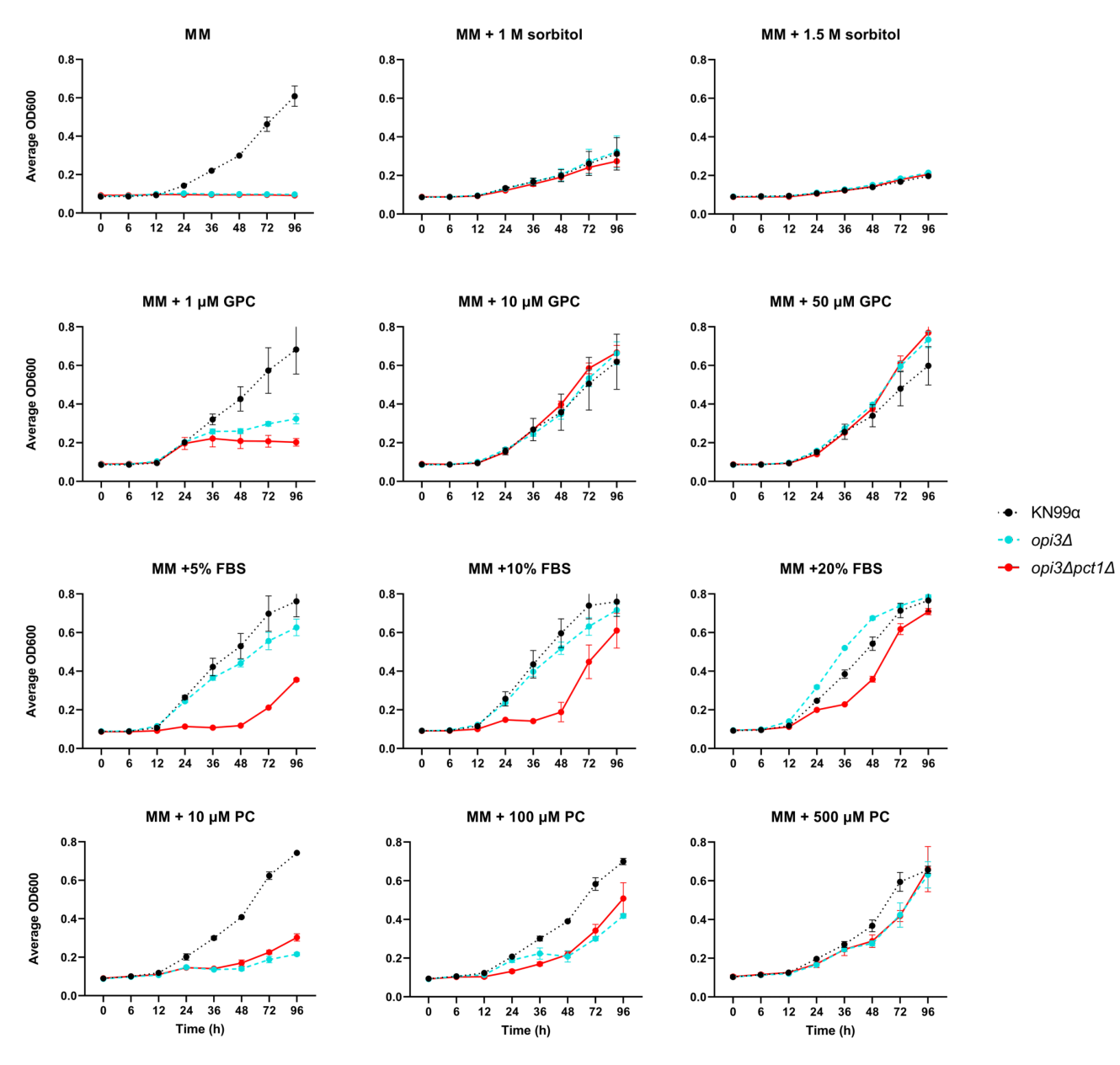


**Supplementary Figure 3. Growth kinetics of KN99α wild type, *opi3Δ* and *opi3Δpct1Δ* mutants in varying concentrations of sorbitol, α-glycerophosphorylcholine (GPC), Fetal bovine serum (FBS) and phosphatidylcholine (PC).** A total of 1 × 10⁴ cells from each strain were inoculated into different media for growth curve analysis, with optical density readings (600 nm) taken every 30 minutes over a period of 96 hours at (A) 30°C and (B) 37°C. The wild-type parental strain KN99α was used as control.


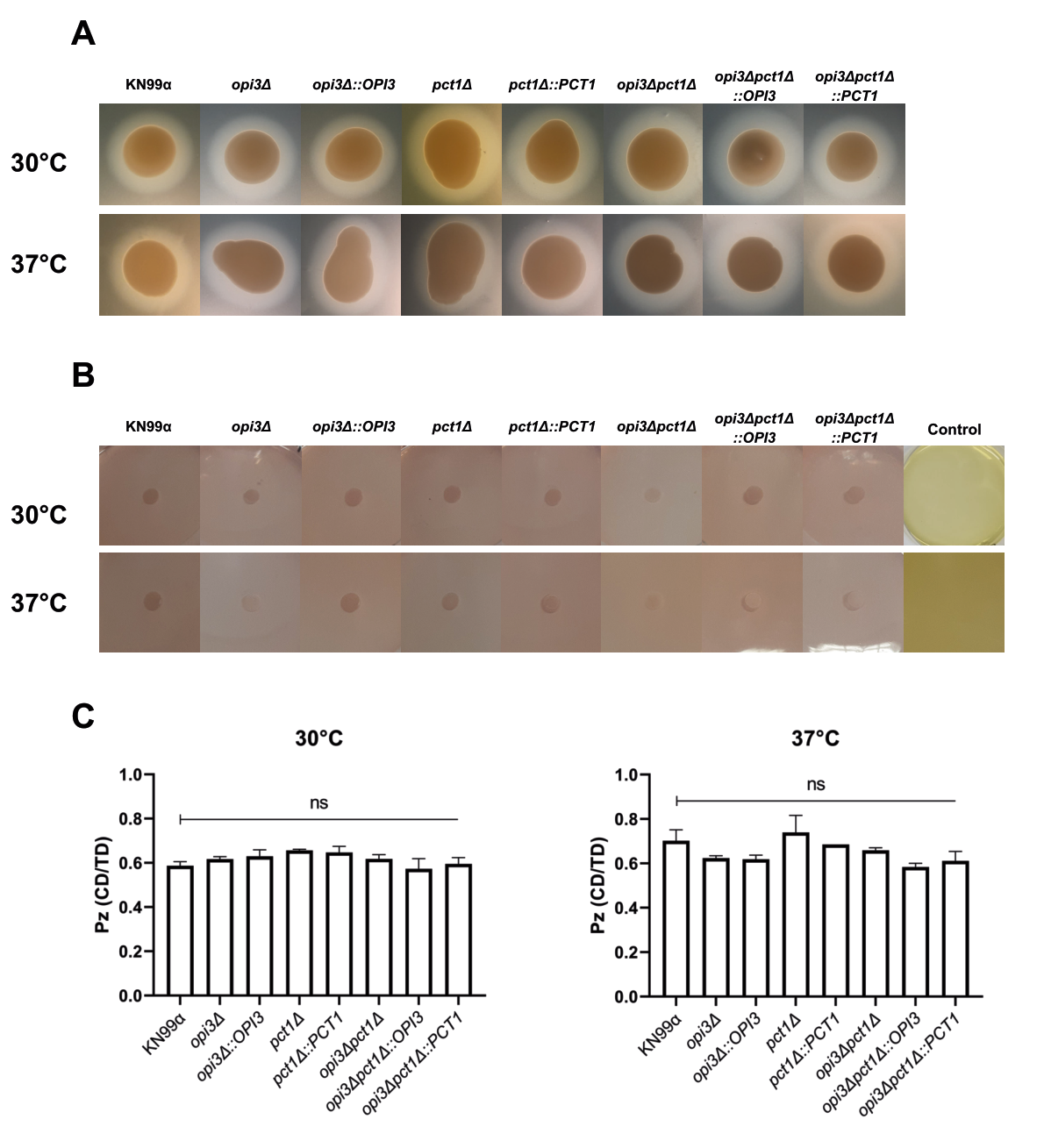


**Supplementary Figure 4. Phospholipase and urease secretion assays with phosphatidylcholine mutant strains and wild type KN99α.** (A) Strains were grown in egg yolk (10%) agar for 72 h at 30°C and 37°C. Precipitation halo around colonies denotes activity of phospholipases confirming secretion. (B) Strains were grown for 48 h at 30°C and 37°C in Christiansen agar which changes color as urea is hydrolyzed into ammonia, leading to an increase in pH. The plates were photographed after 72 hours of growth. The pink/red color indicates strains that secrete urease, while yellow color represents the negative control. (C) After incubation period, phospholipase assay colonies were measured. Pz is the ratio between the colony diameter (DC) and the total diameter including the precipitation zone (DT). Strains positive for phospholipase secretion have a Pz value < 1, while those that do not secrete the enzyme have a Pz value = 1. Two biological replicates were performed, and a one-way ANOVA test was used for statistical analysis of the data.


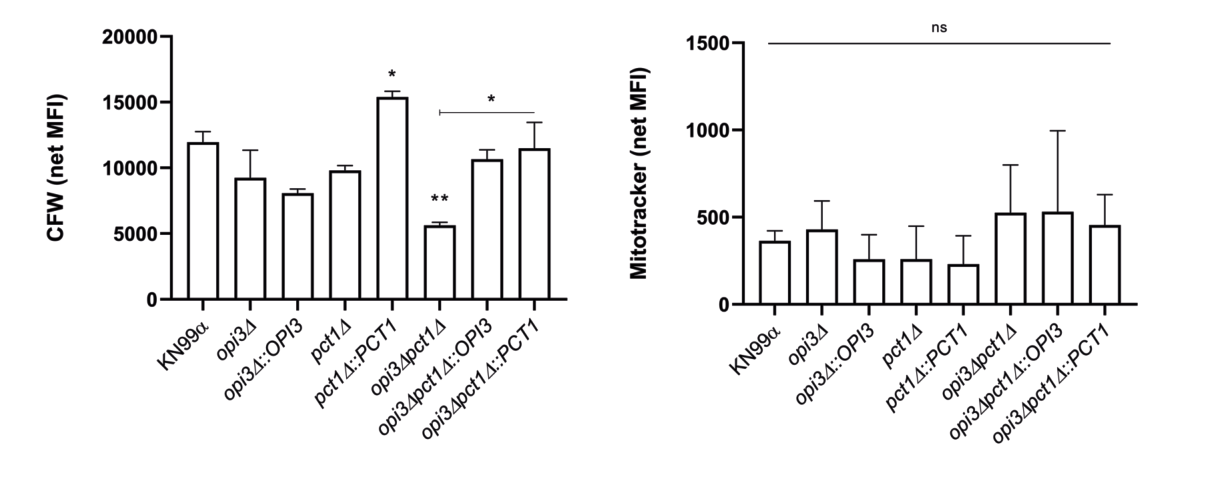


**Supplementary Figure 5. Cytometric analysis of phosphatidylcholine mutant strains and wild type KN99α.** Strains were grown in MM for 15 hours prior to staining with Calcofluor White or MitoTracker Orange CMTMRos for 30 min. Average mean fluorescence intensity (MFI) of 20,000 stained cells normalized to unstained control measured using the PE-Texas Red filter. FlowJo software was used for data analysis and One-way ANOVA test with multiple comparisons was used for statistical analysis. **p* = <0.05; ** = *p* < 0.01





**Supplementary Figure 6. Wild type and phosphatidylcholine mutant strains extracellular vesicles (EVs) characterization.** Strains were grown in liquid YPD for 48 h under agitation at 30°C. Then, cells were washed and a 1x10^5^ yeast/mL inoculum was plated in 100% Sabouraud dextrose agar and incubated for 5 days at 30°C. Yeast cells were then collected with PBS, centrifuged and supernatant was filtered with a 0,45 µm pore filter. EVs were present in the filtered supernatant were then separated by ultrafiltration in an Amicon system with a 100 KDa membrane (Merck Millipore—Burlington, MA, USA) followed by ultracentrifugation. GXM was removed by capture ELISA technique with 18B7 antibody. (A) Dynamic scattering light (DLS) analysis, measuring hydrodynamic diameter of EVs. EV samples were indirectly quantified by (B) protein and (C) ergosterol using the colorimetric assay Micro BCA Protein assay kit for protein quantification and the fluorimetric assays Amplex Red Cholesterol assay kit for ergosterol quantification (both by Thermo Fisher Scientific—Waltham, MA, USA), following the manufacturer’s instructions.

**Suplementary Table 1. Primers used in this study.**

| **Primer** | **Name** | **Target** | **Sequence *5’-3’*** | **Use** |
| --- | --- | --- | --- | --- |
| **1** | LF583 | *PCT1* | TCCAGCCTCTGAGAATAGTT | Double Joint-PCR  Reconstitution |
| **2** | LF584 | *PCT1* | *ATCATGTCATAGCTGTTTCCTG*GCAGTGATAGTATAGAGCTG | Double Joint-PCR |
| **3** | LF20 | *HPH* | CAGGAAACAGCTATGACATGAT | Double Joint-PCR |
| **4** | LF585 | *HPH* | CACAGTTTGCCAGTGATAC | Double Joint-PCR  Internal (*3’*) confirmation of deletion |
| **5** | LF586 | *HPH* | CTGACCTATTGCATCTCCCGC | Double Joint-PCR  Internal (*5’*) confirmation of deletion |
| **6** | LF21 | *HPH* | GTAAAACGACGGCCAGTGC | Double Joint-PCR |
| **7** | LF587 | *PCT1* | GCACTGGCCGTCGTTTTACCTGTACTACTCGCTATCGTT | Double Joint-PCR |
| **8** | LF588 | *PCT1* | GGTGTTTATCCTGAAGTGG | Double Joint-PCR  Reconstitution |
| **9** | LF589 | *PCT1* | CTACCAGAAGCCCTTTAATC | External (*5’*) confirmation of deletion |
| **10** | LF495 | *PCT1* | CCATCTCTCAACGCCATCTTC | Internal (*3’*) confirmation of deletion and reconstitution |
| **11** | LF496 | *PCT1* | TCGACCCTCAGATGTTCCTGT | Internal (*5’*) confirmation of deletion and reconstitution |
| **12** | LF590 | *PCT1* | CTCCCTATCCACACTAACC | External (*3’*) confirmation of deletion |
| **13** | LF575 | *OPI3* | ATTCCCTTCCCTTCCCGTCTA | Reconstitution |
| **14** | LF576 | *OPI3* | CCGAGATAAGTGCCAGTGACA | Internal (*5’*) confirmation of deletion and reconstitution |
| **15** | LF577 | *OPI3* | TTGTACGCCTTTGTTGCCATG | Internal (*3’*) confirmation of deletion and reconstitution |
| **16** | LF578 | *OPI3* | GAGAAATGTCGGTAGGCGGAT | Reconstitution |

*hph*: Hygromycin phosphotransferase (confers resistance to Hygromycin B – HygR)
